# A systematic review of clinical effectiveness and safety for historical and current treatment options for metachromatic leukodystrophy in children, including atidarsagene autotemcel

**DOI:** 10.1186/s13023-023-02814-2

**Published:** 2023-08-29

**Authors:** Nigel Armstrong, Andrew Olaye, Caro Noake, Francis Pang

**Affiliations:** 1 Kleijnen Systematic Reviews Ltd, York, YO19 6FD UK; 2 Orchard Therapeutics, 245 Hammersmith Road, 3rd Floor, London, W6 8PW UK

**Keywords:** Metachromatic leukodystrophy (MLD), Gene therapy (GT), Haematopoietic stem cell gene therapy (HSC-GT), OTL-200, Libmeldy, Atidarsagene autotemcel (arsa-cel), Haematopoietic stem cell transplantation (HSCT), Systematic review

## Abstract

**Objective:**

To understand the benefit-risk profile for historical and current treatments for MLD.

**Methods:**

A systematic review was conducted on the effectiveness, safety, and costs of MLD treatments: allogeneic haematopoietic stem cell transplantation (HSCT) and atidarsagene autotemcel (arsa-cel) according to best practice.

**Results:**

A total of 6940 titles and abstracts were retrieved from the literature searches and 26 from other sources. From these, 35 manuscripts reporting on a total of 12 studies were selected for inclusion in the review. There were no controlled multi-armed trials. However, we provide observations comparing two interventional therapies (alloHSCT and arsa-cel) and each of these to standard/supportive care (natural history). There were no benefits for survival, gross motor function and cognitive function for LI patients receiving alloHSCT, as patients experienced disease progression similar to LI natural history. For juvenile patients receiving alloHSCT, no differences in survival were observed versus natural history, however stabilisation of cognitive and motor function were reported for some patients (particularly for pre- or minimally-symptomatic LJ patients), while others experienced disease progression. Furthermore, alloHSCT was associated with severe complications such as treatment-related mortality, graft versus host disease, and re-transplantation in both LI and EJ treated patients. Most LI and EJ patients treated with arsa-cel appeared to have normal development, preservation, or slower progression of gross motor function and cognitive function, in contrast to the rapid decline observed in natural history patients. A survival benefit for arsa-cel versus natural history and versus alloHSCT was observed in LI patients.LI and EJ patients treated with arsa-cel had better gross motor function and cognitive function compared to alloHSCT, which had limited effect on motor and cognitive decline. No data has been reported for arsa-cel treatment of LJ patients.

**Conclusions:**

Overall, this systematic review indicates that compared to NHx and HSCT, treatment with arsa-cel results in clinically relevant benefits in LI and EJ MLD patients by preserving cognitive function and motor development in most patients, and increased survival for LI patients. Nevertheless, further research is required to confirm these findings, given they are based on results from non-RCT studies.

**Supplementary Information:**

The online version contains supplementary material available at 10.1186/s13023-023-02814-2.

## Background

Metachromatic leukodystrophy (MLD), a rare inherited condition caused by arylsulfatase A (ARSA) deficiency, which results in the accumulation of fats (sulfatides) leading to the destruction of neurons and the protective fatty layer (myelin) surrounding the nerves in the brain and spinal cord [1]. MLD is a progressive disease that results in loss of all previously acquired motor, language, and cognitive skills, dysphagia, seizures, spasticity, and eventually death [2].

There are several subtypes of MLD classified by age at disease onset i.e., late infantile (LI; symptom development from birth to < 30 months of age), juvenile (J; subdivided into early juvenile [EJ; onset from 30 months to < 7 years] and late juvenile [LJ; onset 7 to < 17 years]) and adult onset (onset ≥ 17 years). LI MLD is the most common variant occurring in 50–60% of cases [2]. The clinical course typically begins with a pre-symptomatic phase with normal motor and cognitive development, followed by a period of developmental plateau or the appearance of symptoms and subsequent rapid disease progression ending with premature death of the patient. The initial disease manifestation varies between subtypes with symptoms including abnormal gait, problems with speech, impaired fine motor skills, concentration, and behavioural problems, among others. LI MLD patients experience rapid and homogeneous disease progression after symptom onset resulting in severe motor and cognitive impairment between 2 and 4 years of age [3, 4]. In EJ MLD, disease progression after symptom onset is initially somewhat slower than in LI MLD, but once the ability to walk independently is lost, disease progression is as rapid as that observed in LI MLD. LJ and adult onset MLD often have a more protracted disease course with cognitive and behavioural function affected more than motor function [5].

Besides atidarsagene autotemcel (also referred to as arsa-cel and previously known as compound number OTL-200, trade name Libmeldy™) there are currently no licensed treatment options for MLD; treatment is limited to supportive care (i.e., physiotherapy, muscle relaxants, pain management therapies) aiming to manage disease complications and preserve patients’ health related quality of life (HRQoL). Family and patients can also benefit from counselling sessions [6]. Allogeneic haematopoietic stem cell transplantation (HSCT) has been used as a treatment for MLD, but with limited effect in patients with early-onset MLD (LI and EJ) or those with more advanced symptoms and with better results in LJ or adult MLD patients treated before symptom onset [7, 8].

Atidarsagene autotemcel, a new treatment for MLD, is an ex vivo autologous hematopoietic stem and progenitor cell-based gene therapy that involves extraction of CD34+ stem cells from a patient’s bone marrow or blood, approved in the EU/Norway/Liechtenstein/Iceland and the UK and currently used as an investigational therapy in the US [9]. The stem cells are genetically-modified by a lentiviral vector and following myeloablative conditioning to make space for the genetically-modified cells, returned to the patient by intravenous infusion. The corrected cells can then differentiate and migrate to affected tissues and produce a functional version of the ARSA enzyme. The aim of the treatment is to halt disease progression and/or modify its natural course [1].

The aim of the systematic review was to understand and to summarize the current evidence on the effectiveness and safety of atidarsagene autotemcel, other therapies and standard/supportive care for the treatment of MLD in children (≤ 17 years).

## Methods

The systematic review followed recommendations of the Centre for Reviews and Dissemination (CRD) guidance for undertaking reviews in healthcare [10] and the Cochrane Handbook for Systematic Reviews of Interventions [11]. The study was reported according to the Preferred Reporting Items for Systematic Reviews and Meta-Analyses (PRISMA) guidelines [12]. The review protocol was registered on the PROSPERO database (CRD42020192663) prior to study commencement.

The study included randomized controlled trials (RCTs), prospective or retrospective single arm or cohort studies with > 5 participants and any economic evaluation of patients (age ≤ 17 years) with early-onset pre-symptomatic or symptomatic MLD of any type (LI, J, EJ) or LJ. If populations included mixed age groups, only studies where data was reported separately for those with early-onset MLD (i.e., actual or predicted age of onset < 7 years) were included. Case reports and cross-sectional studies were excluded.

The intervention of interest was atidarsagene autotemcel. The comparators are standard care and HSCT. Standard care could also be described as best supportive or usual care and includes any of the following including their combinations: management of dystonia, infections, seizures, or secretions; pain relief/sedative drugs; feeding support including gastrostomy; psychological and social support including specialist schooling; coordination of the multidisciplinary team and community care; genetic advice and planning; and end of life care). Allogeneic HSCT was also included as a comparator. The effectiveness outcomes of interest included mortality; progressive disease; gross motor function; neurological function; cognitive functions (cognitive impairment and language skills); ARSA activity and HRQoL. The safety outcomes (adverse events [AEs]) are presented separately.

MEDLINE, MEDLINE In-Process, MEDLINE Daily Update, MEDLINE Epub Ahead of Print, PubMed, Embase, Cochrane Central Register of Controlled Trials (CENTRAL), Science Citation Index (SCI), Northern Light Life Sciences Conference Abstracts, WORLD Symposium, National Institutes of Health (NIH) ClinicalTrials.gov and Orphanet Clinical trials Search were searched for relevant studies from database inception to July 2021 without language or publication limits. The MEDLINE search strategy is shown in Additional file 1: Appendix 1. The bibliographies of included research and review articles were checked for additional relevant studies. An additional publication of a comparison between an atidarsagene autotemcel trial (Study 201,222 (NCT01560182) plus additional patients recruited through an Expanded Access Framework (EAF)) and a natural history (NHx) cohort published after the searches was also included [13]. This study will be referred to as Fumagalli et al. [13].

Two reviewers independently screened articles for inclusion according to prespecified inclusion criteria (See Additional file 1: Table S1) at title/abstract and full text stage, assessed quality and performed data extraction. Any discrepancies between reviewers were resolved through consensus or consultation with a third reviewer. Data from the included studies were extracted, stored, and analysed using Microsoft Excel. For each study, the background study information, patient baseline characteristics (e.g., age, MLD symptom status), interventions/study arms compared (description of interventions and comparators), outcomes assessed (e.g., definition of outcome, methods of assessment), results (e.g., numbers, percentages, and effect sizes with confidence intervals [CIs, where relevant]) and follow-up time were extracted. The risk of bias in non-randomised studies was assessed using the Joanne Briggs Institute (JBI) Critical Appraisal Checklist for Non-randomised Experimental Studies [14]. RCTs were to be assessed using the Cochrane Risk of Bias Tool for Randomised Controlled Trials [15]. A narrative summary of all included studies was performed across all types of MLD (pre- or symptomatic; LI, J, EJ or LJ). The data are summarised using text and where relevant, accompanying tables and figures.

## Results

### Study selection and overview of included studies

A total of 6940 titles and abstracts were retrieved from the literature searches and 26 from other sources. From these, full papers were obtained for 197 citations. After further review, 162 papers were excluded.

**Fig. 1:**
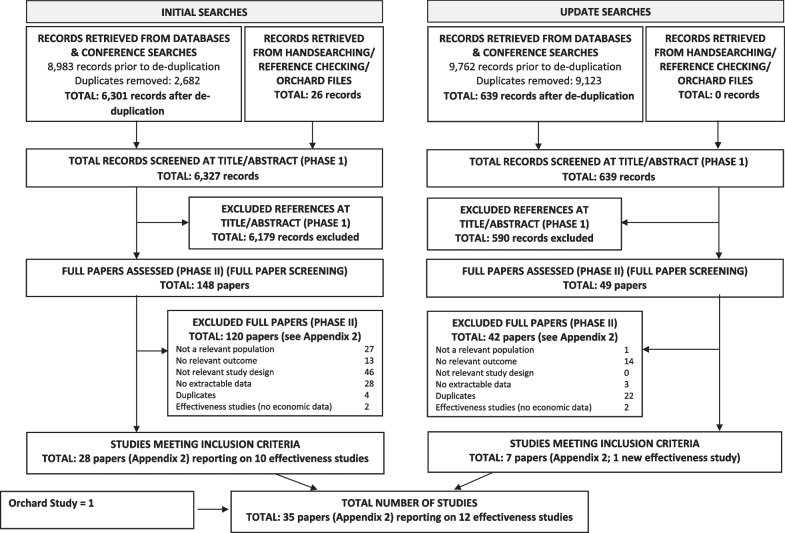
Flow diagram

Thirty-five papers reporting on a total of 12 studies were selected for inclusion in the review. The study selection process is detailed in Fig. 1 (Study flowchart). The list of studies excluded at full paper screening are shown in Additional file 1: Appendix 2 (Tables S11 to S16).

Additional file 1: Table S2 provides details on study designs and Table 1 detail on population characteristics from 13 studies included in the systematic review. The studies were mostly single arm and used retrospective data collection methods. Of those with comparative data, one, Fumagalli et al. [13], compared arsa-cel to NHx, and the other two studies compared HSCT to standard care [16, 17]. Note that Fumagalli et al. [13], whilst having been published in 2022, was based on data that the authors had access to during the conduct of the systematic literature review

**Table 1:** Summary of population characteristics

Study ID (sample size)	Symptom status	MLD type	Baseline ARSA (nmol/hr/mg) in PMBC	Age at diagnosis	Age at symptom onset	Age on treatment initiation/initial assessment	Gender—male/female	Data for population subgroups reported?
Bley 2013 [18] (n = 9)*	Mixed	J	NR	NR	NR	NR	NR	No
Bohringer 2010 [19] (n = 8)*	Mixed	Paediatric	NR	NR	NR	Range 7 months–15 years	NR	No
Boucher 2015 [21] (n = 31)	Mixed	LI to J^a^	Mean 8.84 at treatment (n = 26)	Mean 8.45 years (n = 31)	Range 0.83–26.3 years	Mean 9.05 years	9 (29%)/22 (71%)	LI (n = 4); J (n = 27)
EUROCORD 2018 [22] (n = 60 MLD)	Mixed	LI to J^b^	NR	NR	NR	Median 4.3 years (0.1–22.7 years)	NR	No
Groeschel 2016 [16]* (n = 65)	Mixed	J^a^	NR	NR	Mean 7 years	Median 7 years (1.5–18.2 years)/Mean 8.7 years	33 (50.8%)/32 (49.2%)	No
Kehrer 2014 [26]*^^ (n = 59)	Mixed	LI to LJ^a^^	NR	NR	Median 17 months (9–27 months) LI group (n = 23) Median 76 (32–162 months) J group (n = 36)	NR	32 (54.2%)/27 (45.8%)	LI (n = 23); J (n = 36)
LDM/1 study [25]** (n = 41)	NR/unclear	LI to LJ	NR	NR	< 15 months–14 years	NR	19 (46%)/22 (54%)	LI (n = 22); EJ (n = 14); LJ (n = 5)
Martin 2013 [23] (n = 27)	Mixed	LI to LJ^c^^	NR	Mean 7 years (range 0–16.1 years)	NR	Median 5.2 years	18 (66.7%)/9 (33.3%)	LI (n = 10); J (n = 17)
Fumagalli et al. 2022 (Atidarsagene arm) (n = 29) [13]	Mixed	LI to EJ^d^	NR	NR	NR	Mean (SD) 12.81(4.3) months—LI (n = 16) 65.86 (33.4) months—EJ (n = 13)	16 (55%)/13 (45%)	LI (n = 16); EJ (n = 13)
Fumagalli et al. 2022 (Natural history arm) [13] (n = 31)	Symptomatic at enrolment	LI to EJ^d^	NR	NR	NR	Mean (SD) 20.64 (4.7) months—LI (n = 19) 51.98 (19.2) months—EJ (n = 12)	13 (42%)/18 (58%)	LI (n = 19); EJ (n = 12)
Prasad 2008 [24] (n = 15)	NR/unclear	Paediatric	NR	NR	NR	NR	NR	No
Singh 2012 [20] (n = 11)	NR/unclear	Paediatric^e^	NR	NR	NR	NR	NR	No
van Rappard 2016 [17] (n = 7)	NR/unclear	LI to J^f^	NR	NR	NR	NR	NR	No

The data only considers those patients with paediatric MLD that are eligible for inclusion in this review; data relevant to adult onset and any other diseases are not considered in this summary

If no symbol the authors have not defined ages for the different MLD types

Mixed—a mixture pre-symptomatic and symptomatic patients

Paediatric—patients were children but not reported whether disease was infantile and/or juvenile type

*ARSA* arylsulfatase A, *EJ* early juvenile, *J* juvenile, *LI* late infantile, *LJ* late juvenile, *I* infantile, *n* number of participants, *MLD* metachromatic leukodystrophy, *mth* month, *NR* not reported, *PMBC* peripheral mononuclear blood cells, *SD* standard deviation, *yr* year

*Indicates that there is a possibility of overlap with populations reported in other studies based in German study centres and/or using the LEUKONET database

**Indicated that there is a possibility of overlap of patients between two studies (i.e., LDM/1 study and the natural history study arm of Fumagalli et al. 2022), however, the actual number is unclear

^LJ population is no longer of primary relevance to the proposed treatment indication for Atidarsagene

^^One additional paper was identified during the 2021 update which included more patients (LI: n = 35; EJ: n = 18; LJ: n = 28) and longer follow-up (the actual length was not reported). The overlap between previously reported patients and the new publication is not clearly described in text, thus, the results are included in Appendix 13

^a^Based on following MLD definitions: LI < 30 months; J 30 months–15 years; Adult disease ≥ 16 years

^b^Based on following MLD definitions: LI 0–4 years; EJ 4–6 years; LJ 6–16 years; Adult disease > 16 years

^c^Based on following MLD definitions: LI 0.5-4 years; EJ 4–6 years; LJ 6–16 years); Adult disease > 16 years

^d^Based on the following MLD definitions: LI presence of 2 of the following 3 criteria: Age at onset of symptoms in the older sibling(s) ≤ 30 months; Two null (0) mutant ARSA alleles; Peripheral neuropathy at ENG study (NCV Index < 2 SD below normal range). EJ in the presence of 2 of the following 3 criteria: Age at onset of symptoms in the subject or older sibling(s) between 30 months and 6 years (i.e., had not celebrated 7th birthday); One null (0) and 1 R mutant ARSA allele(s); Peripheral neuropathy at ENG study

^e^Based on the following definition: < 16 years

^f^Based on the following MLD definitions: LI < 30ths; J 2.5–16 years; Adult > 16 years

The prospective arm of Fumagalli et al. [13] aimed to evaluate the clinical efficacy and safety of atidarsagene autotemcel and a (NHx) arm (n = 31) as a historical control. The atidarsagene autotemcel arm consisted of Study 201,222 (n = 20) plus the so-called Extended Access Framework (EAF) (n = 9), which were similar in design and outcomes, allowing combination of the data [13]. Note that atidarsagene autotemcel was not studied in LJ patients and so results would be restricted to the LI and EJ population.

As expected from the rarity of the condition, the studies included low number of patients and population characteristics were poorly reported. Additional file 1: Table S3 summarises the various treatments, which highlights the lack of information on standard care and variation in HSCT implementation. Due to the differences in the definitions of types of MLD across the studies, comparisons between studies are limited. Therefore, if results from comparative studies are available, any other results are included as appendices.

### Risk of bias assessment

The risk of bias in the 12 clinical studies was assessed using the JBI Critical Appraisal Checklist for Non-randomised Experimental Studies and summarised in Table 2. The majority of studies had at least one assessment criterion judged at high risk of bias with only one study with no criterion judged high risk of bias [13]. Overall, twelve studies were judged at high risk of bias and one at an unclear risk of bias [13].

**Table 2:** Summary of risk of bias assessment for non-randomised experimental studies

Study ID	Source	Assessment criterion								
		1	2	3	4	5	6	7	8	9
Bley [18]	Abstract	Yes	NA	NA	No	Unclear	Unclear	NA	Unclear	NA
Bohringer [19]	Abstract	Yes	NA	NA	No	No	Unclear	NA	Unclear	Unclear
Boucher [21]	Full paper	Yes	NA	NA	No	Yes	No	NA	Yes	No
EUROCORD [22]	Full paper	Yes	NA	NA	No	Yes	Unclear	NA	Yes	No
Groeschel [16]	Full paper	Yes	Unclear	Unclear	Yes	Unclear	No	No	Yes	Yes
Kehrer [26]	Full paper	Yes	NA	NA	No	NA	NA	NA	No	Yes
LDM/1 study [25]	Full paper	Yes	NA	NA	No	Unclear	Unclear	NA	Yes	Yes
Martin [23]	Full paper	Yes	NA	NA	No	Yes	Yes	NA	Yes	No
Fumagalli et al. [13]	Full paper	Yes	Yes	Unclear	Yes	Yes	Unclear	Yes	Yes	Yes
Prasad [24]	Full paper	Yes	NA	NA	No	Yes	Unclear	NA	Unclear	No
Singh [20]	Abstract	No	NA	NA	No	No	Unclear	NA	Unclear	Unclear
van Rappard [17]	Full paper	Yes	No	Unclear	Yes	Yes	No	No	Unclear	Unclear

*NA* not applicable, *CUP* compassionate use programme, *HEP* Hospital exemption programme

1: Is it clear what is the cause (intervention) and what is the effect (outcome) (i.e. no confusion about what comes first)

2: Were the participants included in any comparisons similar?

3: Were the participants included in any comparisons receiving similar treatment/care, other than the intervention of interest?

4: Was there a control group?

5: Were there multiple measurements of the outcome both pre and post the intervention?

6: Was follow up complete and if not, were differences between groups in terms of their follow up adequately described and analysed?

7: Were the outcomes of participants included in any comparisons measured in the same way?

8: Were outcomes measured in a reliable way?

9. Was appropriate statistical analysis used?

Studies were poorly reported. Three studies were reported as abstract only [18–20]. Most studies, except Singh 2012 [20], clearly reported the intervention and its effects. A control group was present only in three studies [13, 16, 17]. Six studies reported multiple measurements of the outcome both pre and post intervention [13, 17, 21–24]. Follow-up was incomplete or differences between groups in terms of their follow-up were not adequately described and analysed in three studies. [16, 17, 21] and unclear in seven studies [13, 18–20, 22, 24, 25]. Six studies measured the outcomes in a reliable way [13, 16, 21–23, 25]. An appropriate statistical analysis was used in four studies [13, 16, 25, 26].

### Clinical effectiveness

#### Survival

Eleven studies [13, 16–25] reported data on overall survival including eight studies reporting Kaplan Meier estimates [13, 16, 17, 21–25]. The summary of survival data for patients treated with atidarsagene autotemcel, HSCT or receiving best supportive care is provided in Table 3.

**Table 3:** Summary of survival data

Treatment	MLD type	Symptom status	Time point	n	% survival	Source
Atidarsagene	LI	Pre-symp	Median follow-up 3.0 (1.0–7.5) years	16/16	100	Fumagalli et al. [13]
	LI	Pre-symp	6 years of age	7/16	100%	Fumagalli et al. [13]
	EJ	Mixed	9 years of age	NR/11	75.5	Fumagalli et al. [13]
	EJ	Mixed	10 years of age	NR/11	75.5	Fumagalli et al. 2022 [13]
	EJ	Mixed	11 years of age	NR/11	75.5	Fumagalli et al. [13]
HSCT	Child	NR/unclear	1 year	NR/NR	65	Prasad [24]—P
	Child	NR/unclear	5 year	NR/NR	57	Prasad [24]—P
	Child	Mixed	NR^+^	8/8	100	Bohringer [19]—NR*
	LI to LJ^c^	NR/unclear	Last follow-up (NR)	5/7	72.4	van Rappard [17]—R
	LI to LJ^c^	Mixed	5 year	20/27	74.1 (95% CI 53.2–86.7)	Martin [23]—R
	LI to J	Mixed	NR/unclear	16/31	51.6	Boucher [21]—R
	LI	NR/unclear	1 year	1/2	50	van Rappard [17]—R
	LI	Mixed	5 years	NR/NR	50	Boucher [21]—R
	LI	Mixed	5 years	6/10	60 (95% CI 25.3–82.7)	Martin [23]—R
	LI	Mixed	6 years	NR/NR	59	EUROCORD [22]—R
	EJ	Mixed	6 years	NR/NR	80	EUROCORD [22]—R
	J	NR/unclear	1 year	4/5	80	van Rappard 2016 [17]—R
	J	Mixed	5 years	14/17	82.4 (95% CI 54.7–93.9)	Martin [23]—R
	J	Mixed	5 years	NR/NR	59 (95% CI 38–75)	Boucher [21]—R
	J	Mixed	5 years^++^	19/24	79.2	Groeschel [16]^—^R*
	LJ^c^	Mixed	6 years	NR/NR	79	EUROCORD [22]—R
	J	Mixed	Median 7.5 (3.0–19.7) years	18/24	75	Groeschel [16]—R*
	J	Mixed	NR	6/9	66.7	Bley [18]—R*
Standard care	Child	NR/unclear	At study analysis (NR)	8/11	72.7	Singh [20]—R
	LI to LJ^c^	NR/unclear	Last follow-up (22–93 months)	11/19	57.9	van Rappard [17]—R
	LI	Pre-symp	6 years of age	5/19	70.8 (95% CI 43.6–86.7)	Fumagalli et al. [13]
	LI	NR/unclear	Last follow-up (19–93 months^)	2/6	33.3^a^	van Rappard [17]—R
	LI	NR/unclear	Last follow-up (NR)**	3/16	18.8^d^	LDM/1 study [25]—R/P
	LI	NR/unclear	5 years	NR	56	LDM/1 study [25]—R/P
	LI	NR/unclear	10 years	NR	40	LDM/1 study [25]—R/P
	EJ	Mixed	9 years of age	12/12	100 (95% CI 100–100)	Fumagalli et al. [13]
	EJ	Mixed	10 years of age	11/12	88.9 (95% CI 43.3–98.4)	Fumagalli et al. [13]
	EJ	Mixed	11 years of age	9/12	76.2 (95% CI 33.2–93.5)	Fumagalli et al. [13]
	EJ	NR/unclear	Last follow-up (NR)**	6/9	66.7^e^	LDM/1 study [25]—R/P
	EJ	NR/unclear	5 years	NR	90	LDM/1 study [25]—R/P
	EJ	NR/unclear	10 years	NR	80	LDM/1 study [25]—R/P
	LJ	NR/unclear	Last follow-up (NR)**	4/4	100^f^	LDM/1 study [25]—R/P
	LJ	NR/unclear	5 years	NR	100	LDM/1 study [25]—R/P
	LJ	NR/unclear	10 years	NR	100	LDM/1 study [25]—R/P
	J	Mixed	5 years^++^	41/41	100	Groeschel [16]—R*
	J	NR/unclear	Last follow-up (NR)	9/13	69.2^b^	van Rappard [17]—R

Mixed symptom status refers to population including both pre-symptomatic and symptomatic patients

*EAP* expanded access programme, *I* infantile MLD, *J* juvenile MLD, *EJ* early juvenile MLD, *LJ* late juvenile MLD, *HSCT* haemopoietic stem cell transplantation, *MLD* metachromatic leukodystrophy, *mth* month, *n* number alive, *N* total number analysed, *NR* not reported, *P* prospective study, *Pre-symp* pre-symptomatic, *R* retrospective study, *yr* year

*Indicates that there is a possibility of overlap with populations reported in other studies based in German study centres and/or using the LEUKONET database

**Indicates that there is an overlap between patients with the Fumagalli et al. [13] NHx cohort

*Values in italics are reported to be Kaplan Meier % survival values*

Time point is reported as described by the author(s), where possible the baseline from which time is measured is stated: ^+^After treatment (NR); ^++^After disease onset

^a^Patients died aged 4, 5, 6 and 8 years of age

^b^Patients died aged 8, 11, and 12 (n = 2) years of age

^c^LJ disease is no longer relevant to the indication for Atidarsagene treatment

^d^Thirteen patients died due to disease progression. Sex patients (overall n = 22) were lost to follow-up

^e^Three patients died due to disease progression. Five patients (overall n = 14) were lost to follow-up

^f^One patient (overall n = 5) was lost to follow-up

Five studies reported retrospective or mixed retrospective and prospective data on survival in untreated (natural history) standard care late infantile (LI) to juvenile (J) patients (n = 131) with percentage survival ranging from 18.8% in 16 LI patients (follow-up not reported) to 100% at 5 years in 41 EJ patients.

Eight studies (n = 172) reported survival in patients undergoing HSCT. Approximately 5 years after HSCT survival ranged from 57 to 74.1% regardless of MLD disease subtype [23, 24]. Five to six years after HSCT, survival in LI patients [21–23] ranged from 50 to 60% and in juvenile (J) MLD patients [16, 21, 22, 27] from 59 to 82.4%, with little difference between EJ (80%) and LJ (79%).

One study reported survival in atidarsagene autotemcel treated (n = 29) and NHx patients (n = 31). After a median follow-up of 3.0 years (range 1.0–7.5 years), there were no deaths with seven surviving to age 6 in pre-symptomatic LI MLD patients treated with atidarsagene autotemcel in Fumagalli et al. 2022. In contrast, among the 19 LI MLD NHx patients, five had died by the age of 6 years (70.8% survival). In the EJ population, at the age of 9 years, estimated survival was 75·5% and 100% for the treated and NHx respectively. At 10 and 11 years of age, survival was 75·5% for treated EJ patients compared with 88·9% and 76·2%, respectively for NHx patients.

#### Progressive disease

The number of patients experiencing disease progression was reported by five studies [16, 18–20, 23] and the number of patients dying due to disease progression has been reported by three studies [13, 17, 21]. The summary of disease progression data for patients treated with atidarsagene autotemcel, HSCT or receiving best supportive care is provided in Table 4. Definitions of ‘disease progression’ substantially varied across the studies of HSCT and standard of care.

**Table 4:** Progressive disease by treatment

Treatment	MLD type	Symptom status	Time point	Outcome definition	n/N (%)	Source
Atidarsagene	LI	Mixed	NR/unclear	No. progressed to death	0/16 (0%)	Fumagalli et al. [13]
	EJ	Mixed	8 and 15 months post-treatment	No. progressed to death	2/13 (15%)	Fumagalli et al. [13]
HSCT	NR/unclear	Pre-symp	Post-HSCT	No. remaining asymptomatic	NR/8 (NR%)^c^	Bohringer [19]—NR*
	NR/unclear	Symptomatic	Post-HSCT	No. with progression resulting in neurological disease	NR/8 (NR%)^d^	Bohringer [19]—NR*
	LI to LJ^j^	Mixed	Median 5.1 years (range 2.4–14.7)^+^	No. with significant disease progression	10/27 (37%)	Martin [23]—R
	LI to J	Mixed	1 year	No. progressed to death	2/7 (28.6)^h^	Van Rappard [17]—R
	LI	Mixed	Median 5.1 years (range 2.4–14.7)^+^	No. with significant disease progression	6/10 (60%)	Martin [23]—R
	LI	Mixed	1 year	No. progressed to death	1/2 (50%)^h^	Van Rappard [17]—R
	EJ	Mixed	NR/unclear	No. with further progression of disease	3/3 (100%)^a^	Bley [18]—R*
	J	Mixed	Median 7.5 years (range: 3.0–19.7)^+^	No. long-term surviving patients with SD	11/20 (55%)	Groeschel [16]—R*
	J	Mixed	10 years^+^	No. long-term surviving patients with SD	12/20 (60%)	Groeschel [16]—R*
	J	Mixed	10 years^+^	No. disease progression resulting in a low level of GMF or loss of language	8/20 (40%)	Groeschel [16]—R*
	J	Mixed	Median 7.5 years (range 3.0–19.7)^+^	No. progressed to death	2/24 (8.3%)	Groeschel [16]—R*
	J	Mixed	1 year	No. progressed to death	1/5 (20%)^h^	Van Rappard [17]—R
	J	Mixed	Latest follow-up	No. progressed to death	2/16 (12.5%)^i^	Boucher [21]—R
	J	Mixed	Median 7.5 years (range 3.0–19.7)^+^	No. long-term surviving patients with disease progression	9/20 (20%)	Groeschel [16]—R*
	J	Mixed	Median 5.1 years (range 2.4–14.7)^+^	No. with significant disease progression	4/17 (23.5%)	Martin [23]—R
	LJ^j^	Mixed	Unclear	No. with further progression of disease	0/2 (0%)^b^	Bley [18]—R*
Standard care	NR/unclear	NR/unclear	Mean 4.8 years (3.5–6 years) after first symptom	No. progressed to death	3/11 (27.2%)^e^	Singh [20]—R^g^
	NR/unclear	NR/unclear	Approximately 2 years	No. with disease progression resulting in developmental regression	9/11 (81.8%)^f^	Singh [20]—R^g^
	NR/unclear	NR/unclear	Approximately 5 years	No. with disease progression resulting in developmental regression	11/11 (100%)^f^	Singh [20]—R^g^
	J	Mixed	10 years**	No. long-term surviving patients with SD	13/41 (31.7%)	Groeschel [16]—R*
	J	Mixed	10 years**	No. disease progression resulting in a low level of GMF or loss of language	28/41 (68%)	Groeschel [16]—R*
	J	Mixed	Median 7.5 years (range 3.0–19.7)	No. progressed to death	11/41 (26.8%)	Groeschel [16]—R*

Mixed refers to populations with a mixture of pre-symptomatic and symptomatic patients

*GMF* gross motor function, *I* infantile MLD, *J* juvenile MLD, *EJ* early juvenile MLD, *LJ* late juvenile MLD, *HSCT* haemopoietic stem cell transplantation, *MLD* metachromatic leukodystrophy, *mth* month, *No*. number of patients, *n* number alive, *N* total number analysed, *NR* not reported, *P* prospective study, *Pre-symp* pre-symptomatic, *R* retrospective study, *SD* stable disease, *yr* year

*Indicates that there is a possibility of overlap with populations reported in other studies based in German study centres and/or using the LEUKONET database

Time point is reported as described by the author(s), where possible the baseline from which time is measured is stated: ^+^ After treatment (NR); ** After disease onset

^a^n = 2 received HSCT early during the pre-symptomatic phase, and n = 1 had developed gait disturbance around the time of HSCT. All tested normal in psychodevelopmental tests

^b^One of the two patients had cognitive and motor deficits at the time of HSCT, and the other was symptom-free and remained so for past 10 years

^c^Patients asymptomatic prior to HSCT stayed asymptomatic

^d^Patients who presented with neurological symptoms showed various degrees of progression

^e^3 children died, and 8 children were alive at the time of the study analysis

^f^Most children presented with developmental regression at 2 years of age, but n = 2 manifested with symptoms after 5 years of age

^g^Current age of children at the time of analysis was mean of 10.1 years (range 4.9–20.6); children died and 8 children were alive at the time of the study analysis; mean time in years after first symptom to death was 4.8 years (range 3.5–6)

^h^All were symptomatic patients

^i^Among 16J-MLD patients in our cohort who did not die from transplant-related causes, 2 died from progressive MLD at 8 years and 5 years following initial disease onset

^j^LJ disease is no longer relevant to the indication for Atidarsagene treatment

Ten years after HSCT, eight out of 20 (40%) patients with J MLD had disease progression resulting in a low level of gross motor function or loss of language compared to 28 out of 41 (68%) in non-transplanted J MLD patients. Two out of 24 (8.3%) J MLD children died of rapid MLD progression after HSCT in comparison to 11 out of 41 (26.8%) in an untreated control group [16]. In comparison one out of two (50%) LI MLD and one out of five (20%) J MLD patients died due to disease progression within 1 year of HSCT in another comparator study (n = 7), but outcome data were not reported for the untreated control arm [17].

For patients receiving atidarsagene autotemcel, disease progression was reported mostly within different disease facets, such as brain MRI or neuropsychological outcomes (performance IQ), and result are presented in the sections below. For LI MLD patients treated with atidarsagene autotemcel, there were no progressive disease related deaths at the time of interim analysis (median follow-up of 3 years). At the same timepoint, 2 out of 13 (15%) EJ MLD patients treated with atidarsagene autotemcel died due to disease progression (8- and 15-months post-treatment) [13].

#### Gross motor function

Six studies reported data on gross motor function [4, 13, 16, 17, 21, 25]. Gross motor function was assessed in a variety of ways, but most studies used the Gross Motor Function Measure (GMFM)-88 or the Gross Motor Function Classification (GMFC)-MLD. The summary of gross motor function for patients treated with atidarsagene autotemcel or HSCT versus standard care is provided in Tables 5 and 6 (further non-comparative data on standard care and HSCT are shown in Additional file 1: Tables S4 and S5).

**Table 5:** Gross motor function using GMFM-88 for Atidarsagene versus standard care in Fumagalli et al. [13]

MLD type	Symptom status	Follow-up (years)	Atidarsagene		Standard care		Comparison between OTL and standard care	
			N	% GMFM-88 total score	N	% GMFM-88 total score		
LI	Pre-symp	2	11	73.1	9	7.6	65.6% (95% CI 48.9–82.3)	*p* < 0.001 in favour of Atidarsagene *
LI	Pre-symp	3	10	74.3	12	2.8	71.5% (95% CI 50.3–92.7)	*p* < 0.001 in favour of Atidarsagene*
EJ	Mixed	2	10	78.7	11	36.7	42.0% (95% CI 12.3–71.8)	*p* = 0.036 in favour of Atidarsagene*
EJ	Mixed	3	10	72.9	12	16.3	56.7% (95% CI 33.7%–79.6)	*p* < 0.001 in favour of Atidarsagene*

Mixed refers to populations with a mixture of pre-symptomatic and symptomatic patients

*ANCOVA* analysis of covariance model, *CI* confidence interval, *EJ* early juvenile MLD, *GMFM-88* gross motor function measure-88 items, *LI* late infantile, *LS* least squares, *MD* mean difference, *MLD* metachromatic leukodystrophy; mth month; N total number analysed; pre-symp pre-symptomatic

GMFM-88 consists of 88 questions organised into 5 domains: (a) lying and rolling; (b) sitting; (c) crawling and kneeling; (d) standing; and (e) walking, running, and jumping. Each of the 88 questions is scored from 0 to 3 (maximum number of points = 264). Scores of each dimension are expressed as a percentage of the maximum score for that dimension, and a total score is obtained by averaging the percentage scores across the 5 dimensions, with 0% corresponding to loss of all voluntary movement. The GMFM score is also related to age; by the age of 60 months, most healthy children will achieve their maximum score, approximating 100%

*GMFM-88 data at Year 2 and 3 were analysed using an ANCOVA model fitting age and treatment (Atidarsagene or NHx) and testing the null hypothesis that the difference was 10%. Age was fitted in months for the LI group

**Table 6:** Gross motor function (GMFM-MLD score or GMFC-MLD) for HSCT versus standard care

Treatment	MLD type	Symptom status	Time point	Outcome definition	n/N (%)	Source
HSCT	LI	Mixed	Last available follow-for each patient^	No. with worsening GMFM score	2/2 (100%)	Boucher [21]—R
	LI	NR/unclear	Last available follow-for each patient^	No. with worsening GMFM score	2/2 (100%)	van Rappard [17]—R
	LI to J	Mixed	Last available follow-for each patient^	No. with worsening GMFM score	13/16 (81.25%)	Boucher [21]—R
	J	NR/unclear	Last available follow-for each patient^	No. with worsening GMFM score	2/5 (40%)	van Rappard [17]—R
	J	Mixed	Last available follow-for each patient^	No. with worsening GMFM score	11/14 (78.6%)	Boucher [21]—R
	J	NR/unclear	Last available follow-for each patient^	No. with no change in GMFM score	3/14 (21.4%)	van Rappard [17]—R
	J	Mixed	10 years**	Progression to GMFC-MLD level 5^a^	8/20 (40%)	Groeschel [16]—R*
Standard care	LI	NR/unclear	Last available follow-for each patient^	No. with no change in GMFM score	2/6 (33.3%)	van Rappard [17]—R
	LI	NR/unclear	Last available follow-for each patient^	No. with worsening GMFM score	4/6 (66.7%)	van Rappard [17]—R
	J	NR/unclear	Last available follow-for each patient^	No. with no change in GMFM score	12/12 (100%)	van Rappard [17]—R
	J	Mixed	10 years**	Progression to GMFC-MLD level 5^a^	28/41 (68.29%)	Groeschel[16]—R*

Mixed refers to populations with a mixture of pre-symptomatic and symptomatic patients

*GMFM* gross motor function measure, *I* infantile MLD, *J* Juvenile MLD, *LI* late infantile MLD, *HSCT* haemopoietic stem cell transplantation, *MLD* metachromatic leukodystrophy, *mth* month, *No*. number of patients, *n* number with outcome, *N* total number analysed, *NR* not reported, *P* prospective study, *R* retrospective study, *SD* stable disease, *yr* year

*Indicates that there is a possibility of overlap with populations reported in other studies based in German study centres and/or using the LEUKONET database

Time point is reported as described by the author(s), where possible the baseline from which time is measured is stated: ^After treatment; **After disease onset

^a^GMFC-MLD Level 5 corresponds to ‘only head control possible’

Fumagalli et al. [13] showed a large and statistically significant difference in GMFM-88 in favour of atidarsagene autotemcel compared with NHx patients. Across both MLD subtypes (LI and EJ), patients receiving atidarsagene autotemcel showed better gross motor function at to 3 years follow-up when compared to age-matched NHx patients (Table 5). In contrast, only J type patients receiving HSCT showed any retention of gross motor function vs. non-transplanted (standard care) patients, and in one study the number of patients with no change in GMFM was higher with standard care (100% vs. 21.4%) (Table 6) [21]. Those with LI disease all had declining function (Table 6) [17].

#### Cognitive function—cognitive impairment and language skills

Five studies reported data on cognitive impairment [13, 17, 21, 23, 26] and six studies on language skills [16, 21, 23, 26, 28, 29]. Because very few data were reported for atidarsagene autotemcel the summary of results for cognitive function for patients treated with HSCT or receiving standard care are provided in Additional file 1: Tables S6–S9.

In general, patients with cognitive impairment at baseline appeared to continue to experience a decline in function after HSCT [21], although some patients (n = 5/14) [23] with borderline or delayed cognitive skills at baseline were reported to continue to gain cognitive skills initially (see Additional file 1: Table S8).

It was reported from Fumagalli et al. 2022 that age-equivalent scores showed normal cognitive skills in 20 of 25 (80%) atidarsagene autotemcel treated patients at similar chronological ages at which NHx patients showed severe cognitive impairment [13].

#### Intelligence quotient (IQ)

Three studies reported data on IQ scores [16, 17, 21]. The method of IQ assessment was reported in all except one study [17] with the Wechsler Preschool and Primary Scale of Intelligence (WPPSI) and the Wechsler Intelligence Scale for Children (WISC) scales most commonly used. There were no atidarsagene autotemcel studies reporting IQ, although the cognitive age-equivalent scores measured in Fumagalli et al. 2022 are based on the Development Quotient, which is itself derived from IQ [13].

One study of HSCT versus standard care reported that 11/24J MLD patients were more likely to have an IQ of at least 85 when compared with untreated standard care patients [16]. Another study comparing HSCT versus standard care [17], reported that 46.2% of transplanted patients (n = 7; including n = 2 LI and n = 5 J; mean follow-up 4.7 years) did not experience IQ decline (defined as decrease of at least 6 points), whereas all standard care patients (n = 10; mean follow-up 4.6 years) showed a decline in IQ. However, this study also reported that patients with an IQ score below 75 showed no benefit from HSCT [17]. One study of HSCT also reported that there was a trend across all patients (and MLD subtypes) for a gradual decline in VIQ scores (subscore of WISC) after transplant [21].

#### Neurological function—nerve conduction velocity (NCV) and magnetic resonance imaging (MRI)

Three studies reported data on neurological function assessed using nerve conduction velocity (NCV) [13, 21, 23] and five studies on neurological function assessed using brain magnetic resonance imaging (MRI) [13, 16, 17, 21, 23]. NCV was measured using electroneurography and generally reported using NCV index scores. Brain MRI was used to measure the progression of white matter demyelination and atrophy in the central nervous system and the predominant measure used was the Loes score (Fumagalli et al. [13] used a modified version as described in Sessa et al. 2016). The summary of results for neurological function for patients treated with atidarsagene autotemcel, or HSCT versus standard care is provided in Tables 7, 8, 9 and 10. Only non-comparative NCV data were available for HSCT (see Table 8) (further non-comparative MRI data on HSCT are shown in Additional file 1: Table S10).

**Table 7:** NCV for Atidarsagene versus standard care in Fumagalli et al. [13]

MLD type	Symptom status	Follow-up (years)	Atidarsagene		Standard care		Comparison between OTL and standard care	
			N	Mean NCV index	N	Mean ncv index		
*Total NCV score*								
LI	Pre-symp	2	9	− 7.6	10	− 13.3	Treatment difference 5.8 (95% CI 2.4–9.1	*p* = 0.004 in favour of Atidarsagene
LI	Pre-symp	3	6	− 8.3	10	− 11.5	Treatment difference 3.2 (95% CI 1.0–5.3)	*p* = 0.010 in favour of Atidarsagene

Mixed refers to populations with a mixture of pre-symptomatic and symptomatic patients

*ANCOVA* analysis of covariance model, *CI* confidence interval, *EJ* early juvenile ML, *LI* late infantile, *LS* least squares, *MD* mean difference, *MLD* metachromatic leukodystrophy, *mth* month, *N* total number analysed, *pre-symp* pre-symptomatic

**Table 8:** NCV for HSCT patients

MLD type	Symptom status	Follow-up timepoint after HSCT	Outcome definition	n/N (%)	Source
LI	Mixed	NR	No. with worsening NCV^	1/3 (33.3%)~	Boucher [21]—R
LI	Mixed	NR	No. with worsening NCV*	5/8 (62.5%)	Martin [23]—R
J	Mixed	NR	No. with worsening NCV^	10/15 (66.7%)~	Boucher [21]—R
J	Mixed	NR	No. with worsening NCV *	4/12 (33.3%)	Martin [23]—R
LI	Mixed	NR	No. with stabilised NCV^	2/3 (66.7%)~	Boucher [21]—R
LI	Mixed	NR	No. with NCV stabilised*^	3/8 (37.5%)	Martin [23]—R
J	Mixed	NR	No. with stabilised NCV^	5/15 (33.3%)~	Boucher [21]—R
J	Mixed	NR	No. with NCV stabilised*^	8/12 (66.7%)	Martin [23]—R

*LI* late infantile MLD, *J* juvenile MLD, *HSCT* haemopoietic stem cell transplantation, *MLD* metachromatic leukodystrophy, *NCV* nerve conduction velocity, *n* number of patients with the outcome, *N* total number of patients assessed, *NR* not reported, *P* prospective, *R* retrospective

^Not defined

^~^Data were only reported in a figure from these results were calculated

**Table 9:** MRI Severity (Loes) scores for Atidarsagene versus standard care in Fumagalli et al. [13]

MLD type	Symptom status	Follow-up (years)	Atidarsagene		Standard care		Comparison between atidarsagene and standard care	
			N	LS mean change in MRI Loes from baseline (SD)*	N	LS mean change in MRI Loes from baseline (SD)*		
LI	Pre-symp	2	9	2.4	15	15.3	MD − 12.9 (95% CI − 16.2 to − 9.7)	*p* < 0.001 in favour of Atidarsagene versus standard care control
LI	Pre-symp	3	8	3.6	9	21.7	MD -18.1 (95% CI − 21.1 to − 15.0)	p < 0.001 in favour of Atidarsagene versus standard care control
EJ	Mixed	2	10	9.4	11	17.9	MD − 8.5 (95% CI − 14.7 to − 2.3)	*p* = 0.010
EJ	Mixed	3	9	10.1	12	20.5	MD − 10.4 (95% CI − 17.0 to − 3.8)	*p* = 0.004 in favour of Atidarsagene versus standard care control

Mixed refers to populations with a mixture of pre-symptomatic and symptomatic patients

*ANCOVA* analysis of covariance model, *CI* confidence interval, *EJ* early juvenile MLD, *LI* late infantile, *LS* least squares, *MD* mean difference, *MLD* metachromatic leukodystrophy, *mth* month, *pre-symp* pre-symptomatic, *N* total number analysed

*LS mean calculated using ANCOVA model fitting age and treatment (Atidarsagene or NHx). Age was fitted in mths for the LI group

**Table 10:** MRI Severity (Loes) scores for HSCT versus standard care reported in van Rappard [17]

MLD type	Symptom status	Follow-up (years)	HSCT		Standard care	
			N	Median MRI Loes score (range)*	N	Median MRI Loes score (range)*
LI	NR/unclear	Baseline	2	Median 3 (range 2–3)	6	Median 11 (range 4–17)
LI	NR/unclear	Follow-up^	2	Median 14 (range 8–20)	0	NA
LI	NR/unclear	Baseline	7	Median 6 (range 0–20)	19	Median 15 (range 4–22)
LI	NR/unclear	Follow-up~	7	Median 11 (range 0–25)	4	Median 23 (range 19–26)
J	NR/unclear	Baseline	5	Median 8 (range 0–20)	13	Median 17 (range 12–22)
J	NR/unclear	Follow-up^#^	5	Median 10 (range 0–25)	4	Median 23 (range 19–26)

Higher scores indicate a deterioration in neurological function as assessed by MRI; baseline = pre-HSCT or at diagnosis for standard care; follow-up = post-HSCT or at latest assessment for standard care

Mixed refers to populations with a mixture of pre-symptomatic and symptomatic patients

*ANCOVA* analysis of covariance model, *CI* confidence interval, *EJ* early juvenile MLD, *LI* late infantile, *LS* least squares, *MD* mean difference, *MLD* metachromatic leukodystrophy, *mth* month, *pre-symp* pre-symptomatic, *N* total number analysed, *NA* not applicable

*Not reported in the paper but calculated from individual patient data reported in the paper

^Average follow-up 35 months (range 10–60)

^~^Average follow-up 60 months (range 10–127)

^#^Average follow-up 69 months (range 11–127)

Fumagalli et al. [13] compared NCV scores of LI MLD patients treated with atidarsagene autotemcel with age matched natural history (standard care) control patients [13]. For patients with LI MLD, treatment differences in NCV index scores favoured atidarsagene autotemcel versus standard care control at both 2 years (5.8, 95% CI 2.4–9.1; *p* = 0.004) and 3 years (3.2; 95% CI 1.0–5.3; *p* = 0.010) post treatment (see Table 7).

Two studies of HSCT [21, 23] reported only the numbers of patients with worsening and stabilising NCV, hampering any comparison with data from the atidarsagene autotemcel studies. For both LI and J MLD subtypes, results showed that, depending on the study, between one and two thirds of patients tend to show worsening NCV after HSCT, whilst the remaining patients showed stabilised NCV (see Table 8).

With respect to MRI, HSCT and standard care studies [16, 17, 21, 23] mostly reported the number of patients with improved, stable, or deteriorating MRI scores, hampering comparisons with the atidarsagene autotemcel studies (see Additional file 1: Table S6). Patients undergoing HSCT appeared to show stabilisation and/or improvement in brain MRI after transplantation. In one study of LI and EJ MLD patients [21], 6/11 (54.5%) and 7/9 (77.8%) of HSCT patients showed the same level of demyelination as the previous timepoint/baseline value 1 and 2 years (respectively) after transplantation. Another study [23] reported that 16 out of 19 children (84.2%) showed an improvement in MRI total scores after transplantation.

Fumagalli et al. [13, 29] suggested that atidarsagene autotemcel may stabilise and prevent MRI deterioration in both LI and EJ patients, when compared to untreated natural history (standard care) patients. For patients with LI MLD, treatment differences in brain MRI (Loes) total scores favoured atidarsagene autotemcel over a natural history (standard care) control patients at both 2 years (− 12.9, 95% CI − 16.2 to − 9.7; *p* < 0.001) and 3 years (− 18.1, 95% CI − 21.1, − 15.0; *p* < 0.001) post atidarsagene autotemcel treatment (see Table 9). Corresponding treatment differences in EJ patients compared to standard care were − 8.5, 95% CI − 14.7 to 2.3 (*p* = 0.010) at 2 years, and − 10.4, 95% CI − 17.0 to − 3.8 (*p* = 0.004) favouring atidarsagene autotemcel treatment) at 3 years post atidarsagene autotemcel treatment.

In one study [17], standard care (untreated) LI to J patients showed a deterioration (increase) in MRI Loes scores compared to patients undergoing HSCT. Similarly, non-transplanted J MLD standard care patients had significantly higher MRI severity scores compared to transplanted patients (*p* = 0.06) and a significant increase in severity scores from early to late disease stage (*p* < 0.001), compared to transplanted patients pre- to post-HSCT.

#### ARSA activity

Three studies reported data on ARSA activity [13, 19, 21].

Data on ARSA activity after HSCT (n = 39) was limited and was not reported as mean change from baseline hampering any comparison with atidarsagene autotemcel treated patients. However, one retrospective study [21] reported that at last follow-up (mean 3.5 years post-HSCT) 2/3 (66.7%) of LI patients and 17/24 (63.0%) of J patients (63.0%) had ‘100% ARSA activity’ (no details reported).

All atidarsagene autotemcel treated patients in Fumagalli et al. 2022 showed ARSA activity in peripheral blood mononuclear cells within or above normal range from 3 months post-treatment onward, which was significantly increased above baseline 2 years post-treatment by a mean 18·sevenfold (95% confidence interval CI 8.3–42.2; *p* < 0.001) and 5·sevenfold (95% CI 2.6–12.4; *p* < 0.001) in late-infantile and early-juvenile patients, respectively. Mean ARSA activity in cerebrospinal fluid (CSF) was above the level of quantification by the first post-baseline measurement at 3 months post-treatment, reached normal levels by 6–12 months, and remained within normal range throughout available follow-up (Year 3 early-juvenile; Year 5 late-infantile).

#### Health-related quality of life (HRQoL)

Data on the HRQoL of patients with MLD was very limited and only one study assessing HSCT reported data for mix of LI and J patients [21]. There was no assessment of the change in HRQoL from baseline (pre-treatment) and long-term follow up (duration unclear) was measured using the Cornell-Brown Scale that is often used in dementia patients [30]. There were no atidarsagene autotemcel studies reporting IQ.

Amongst the included patients with evaluable data (n = 12) the Cornell-Brown Scale numerical scores were mostly greater than zero suggesting a favourable HRQoL after HSCT. The only LI MLD patient had a score of 4; the mean score across nine J MLD patients was 10.2 (range − 13 to 23), with only one patient having a negative score (− 13).

#### Safety

Six included studies reported on AEs [13, 16, 17, 19, 21, 23]. Rates of fatal AEs for HSCT are shown in Table 11.

**Table 11:** Number of patients experiencing fatal AE after HSCT

Treatment	MLD type	Symptom status	Timepoint after treatment	n/N (%)	Source—design
HSCT	J	Mixed	NR	13/27 (48.1%)^a^	Boucher [21]—R
	J	Mixed	NR	0/8 (0%)	Bohringer [19]—NR*
	LI	Mixed	NR	2/4 (50%)^b^	Boucher [21]—R
	J	Mixed	Within wks of HSCT (exact time NR)—TRAE	4/24 (16.7%)^c^	Groeschel [16]—R*
	LI to LJ**	Mixed	Median 5.1 years (range 2.4–14.7 years)	7/27 (25.9%)^d^	Martin [23]—R

Mixed refers to populations with a mixture of pre-symptomatic and symptomatic patients

*EJ* early juvenile, *HSCT* haematopoietic stem cell transplant, *LI* late infantile, *n* number with outcome, *N* total number assessed, *NR* not reported, *Pre-symp* pre-symptomatic, *R* retrospective study, *TRAE* treatment related adverse event, *yr* year

*Indicates that there is a possibility of overlap with populations reported in other studies based in German study centres and/or using the LEUKONET database

**LJ disease is no longer relevant to the indication for Atidarsagene treatment

^a^Cause of death: hepatic veno-occlusive disease (VOD) n = 1, cGVHD n = 2, aGVHD n = 1, sepsis n = 5, MLD n = 2, multi-system organ failure (MSOF) n = 1, thrombotic thrombocytopenic purpura (TTP) n = 1

^b^Cause of death: unknown n = 1, hepatic veno-occlusive disease (VOD) n = 1

^c^Transplantation-related mortality occurred in 4 children (17%), all of whom died of infections associated in part with graft rejection

^d^Includes: multiple organ failure n = 1; Respiratory failure (after chronic lung disease) n = 2; Viral infections/malignancy n = 3 (includes one EBV PTLD); Disease progression and infection n = 1

Five studies reported limited data mostly relating to the number of fatal AE after HSCT [16, 17, 19, 21, 23]. In Boucher 2015 [21] 17/48 (35.4%) patients experienced fatal events after HSCT (follow-up not reported) including 13 J MLD patients (hepatic veno-occlusive disease n = 1; Graft versus Host Disease (GvHD) n = 2; acute GvHD n = 1; sepsis n = 5; MLD progression n = 2; multi-system organ failure n = 1; and thrombotic thrombocytopenic purpura n = 1) and two LI MLD patients (cause unknown n = 1; and hepatic veno-occlusive disease n = 1). Treatment related fatal events within weeks post HSCT were experienced by four out of 24 (16.7%) J MLD patients in Groeschel [16]; all died of infections including two with bacterial, infections, one invasive fungal infection and one viral interstitial pneumonitis associated in part with graft rejection. Martin [23] reported that a median of 5.1 years after HSCT 25.9% (7/27) of patients experienced a fatal AE; causes of death included multiple organ failure (n = 1); respiratory failure after chronic lung disease (n = 2); viral infections/malignancy (n = 3); and disease progression and infection (n = 1). Bohringer [19] reported no case of transplant-related mortality or no chronic GvHD.

In Fumagalli et al. [13] all 39 atidarsagene autotemcel treated patients experienced at least one Grade 3+ adverse event: three were fatal (two AEs of dysphagia due to disease progression, one AE of ischaemic stroke, all unrelated to treatment) and the only treatment-related adverse event was the transient development of anti-ARSA antibodies in four patients, which was reported to have not impacted clinical outcomes.

## Discussion

### Summary of findings

To our knowledge, this systematic review is the first to evaluate treatments (atidarsagene autotemcel and HSCT) for children (age ≤ 17 years) with MLD and to compare it to the natural progression of the disease. The previous systematic review by Musolino [31] focused on the effects of HSCT in the treatment of patients with leukodystrophies. The authors searched the literature up to June 2012 and included evidence beyond the scope of this review (case reports, case series and other). Out of 698 patients included in the review, only 114 were MLD patients with the results focusing on safety and the characteristics of HSCT procedures. The systematic review by Mahmood [32], reported alongside the case report of triplets with MLD, identified studies reporting on new cases with definite diagnosis of MLD up to June 2006. Any study reporting exclusively on patients receiving transplants were excluded. The authors focused on patients’ survival.

Overall, this is the first systematic review of atidarsagene autotemcel, HSCT and standard care control patients in MLD and confirmed that the condition is associated with significant mortality and morbidity in children and adolescents. Comparability between treatments was hampered by lack of randomised controlled trials, which is typical for rare diseases. In LI patients, survival for atidarsagene autotemcel appeared longer than for NHx and HSCT as observed in other studies. Survival for EJ patients appeared to be similar between atidarsagene autotemcel treated, HSCT recipients and the NHx cohort. However, no LI and only 15% of EJ patients treated with atidarsagene autotemcel were observed to have died due to disease progression over about 3 years, in contrast to 50% of LI and 20% of J patients treated with HSCT within 1 year [13, 16, 17]. Gross motor function was improved with atidarsagene autotemcel versus with no treatment for both LI and EJ, which contrasted with HSCT, where fewer J patients and no LI patients retained function [21]. HSCT seemed to make little difference to cognitive decline [21], whereas normal acquisition of cognitive skills in the majority of patients with atidarsagene autotemcel treatment [13]. HSCT appeared to be associated with treatment related deaths [16, 17, 19, 21, 23], unlike atidarsagene autotemcel, where no treatment related deaths were observed [13].

### Strengths and limitations

The content of any systematic review is dependent on the methods used and quality of the included research. The review was prepared according to the systematic review methodologies recommended by the Cochrane Collaboration. This review used the best available evidence found through extensive literature searches and other sources with no restrictions.

MLD is a rare genetic disease which poses great challenges to researchers in terms of patient recruitment and comparing treatments to relevant control arms. Most of the studies included in the review had small sample size and included only retrospective, single arm data without control group. A sample size of at least 5 patients was considered sufficient to reduce uncertainty and ensure generalizability of results.

Given the limited patient population and the poorly reported population characteristics, there is potential for overlap of study populations across studies. This is particularly a concern for those studies based in populations from Germany and those using data from the LEUKONET database [16, 18, 19, 26]. Moreover, the data were reported differently across the studies depending on whether patients were assessed from disease onset, diagnosis or with regard to age, making any comparisons between studies difficult.

Overall, outcomes were not well defined across studies. The section ‘progressive disease’ reports on studies that assessed outcome described as ‘progressive disease’ or patients dying with ‘progressive disease’ only. Disease progression has also been reported within different disease facets, such as gross motor function, brain MRI or neuropsychological outcomes (performance IQ).

All included studies were judged as high or unclear risk of bias with poor reporting of the results. Again, small number of patients, and lack of control arm for some studies, limits the reliability of findings. The comparisons between atidarsagene autotemcel, HSCT and standard care is also limited by high heterogeneity in study populations, follow-up time and outcome measures. Due to improvements in HSCT technologies and standard of care of patients with MLD, the results of the studies might not be relevant to current practice. Moreover, studies of HSCT used different sources of stem cells e.g., umbilical cord cells [17, 24] or bone marrow [19]. The comparison with atidarsagene autotemcel studies is limited as this technology involved bone marrow and blood and was not yet applied to all sources of stem cells i.e., umbilical cord cells.

No quantitative analysis was possible due to the lack of comparability of the evidence.

### Research recommendations

The authors identified several gaps in the evidence base for MLD treatments. Firstly, there is lack of prospective studies comparing atidarsagene autotemcel with HSCT. The available evidence does not focus on patient and caregiver HRQoL. Future studies should focus on methodological rigour with more emphasis on the methods of data collection, measurement of outcomes, and the reporting of baseline patient characteristics to ensure the reliability of the results. Any potential of overlap between patient populations in similar studies should be clearly stated and references to relevant studies provided. To aid any future meta-analyses, an agreed set of standardised outcomes measured across all MLD studies should be available: currently, high heterogeneity between studies and outcomes reported hampers any comparisons. An adequate follow-up of MLD patients should also be clearly described.

## Conclusions

Consistent with other research into treatments for rare diseases, there is limited low-quality evidence on which to base an assessment of the clinical effectiveness and cost effectiveness of treatments for MLD in children. Heterogeneity between studies with respect to study designs, populations, follow-up, and outcome measures limits any comparison, along with a lack of direct concurrent control data. Studies were generally small and reliant on retrospective data, particularly with respect to HSCT and standard of care patients. However, studies of the natural history and standard care of the disease in children and adolescents showed that MLD has significant effects on patient mortality and morbidity, including debilitating effects on cognitive and gross motor function. Evidence of the effects of HSCT were unclear especially in EJ patients, and longer-term follow-up suggested that any improvement or stabilisation in cognitive and gross motor function was not likely to be maintained. Data suggested that HSCT may only show potential benefits in LJ. Initial data on patients treated with atidarsagene autotemcel, albeit in only one trial plus EAF, showed promising results particularly when used in patients treated before symptoms appear (pre-symptomatic). This included apparent improvements in survival, as well as reduced rate of decline in cognitive function, and gross motor function. The treatment also appeared well tolerated with no serious or treatment related effects; adverse effects observed appeared mainly due to pre-treatment procedures (conditioning treatment) and MLD disease progression. However, further data is required to confirm these findings and further follow-up of existing studies is ongoing.

### Supplementary Information

**Additional file 1**. Supplementary appendices.

## Data Availability

The datasets used and/or analysed during the current study are available from the corresponding author on reasonable request.
