# Supplementary material for: A systematic review of clinical effectiveness and safety for historical and current treatment options for metachromatic leukodystrophy in children, including atidarsagene autotemcel
Source: Orphanet J Rare Dis. 2023 Aug 29;18:248. doi: 10.1186/s13023-023-02814-2 (PMC10466877; doi:10.1186/s13023-023-02814-2)
Supplement: Supplementary file 1 — Additional file 1. Supplementary appendices. [file 13023_2023_2814_MOESM1_ESM.docx]

### Table 1: Inclusion/exclusion criteria

| **PICOS** | **Description** |
| --- | --- |
| **Population** | Patients with early-onset metachromatic leukodystrophy (MLD), i.e. diagnosed aged ≤ 17yrs were eligible for inclusion.  Subgroups of interest within the main population (data to be reported separately, where available) included:   - Symptomatic MLD - Pre-symptomatic MLD - Late-Infantile MLD - Juvenile MLD - Early-Juvenile - Late-Juvenile*   Where populations included mixed a mixed age group including patients with onset of disease >17yrs, studies were only included if data were reported separately for those with early-onset disease (i.e. symptoms appearing ≤ 17yrs). |
| **Intervention of interest** | The intervention of interest was ex-vivo autologous lentiviral gene therapy specifically:   - OTL-200 (Libmeldy)   The following were included:   - OTL-200 treatment arms in single arm studies - OTL-200 treatment arms in RCTs and cohort studies making a comparison with a relevant comparator treatment of interest (see below). |
| **Comparators of interest** | Comparator treatments of interest were:   - Standard care/best supportive care/usual care** - Allogeneic hematopoietic stem cell transplantation (HSCT)   The following were included:   - Comparator treatment arms in single arm studies - Comparator treatment arms in RCT and cohort studies comparing the comparator treatments with each other or against the intervention of interest (i.e. OTL-200) |
| **Outcome** | Studies must have report at least one of the following specific outcomes which are relevant to the NICE scope[^1^](#_ENREF_1)^,^ [^2^](#_ENREF_2) (also based on outcomes from OTL-200 clinical studies[^3^](#_ENREF_3)^,^ [^4^](#_ENREF_4)):  *Mortality*:  Overall survival (OS) expressed as a hazard ratio (HR), median time to event, or proportion (n/N; %) of patients surviving (if only number of deaths are reported this will be used to calculate the number surviving where possible)  *Progressive disease*:   - Proportion (n/N; %) of individuals with progressive disease (PD) - Median (range) time to progressive disease (PD)   *Motor function*:   - Proportion (n/N; %) of individuals with severe motor impairment - Median (range) time to severe motor impairment - Mean (SD)/median (range) age at time of severe motor impairment - Mean change (SD) from baseline in motor function measured using the following tools:   - Gross Motor Function Classification System (GMFCS)   - Gross Motor Function Measure (GMFM)   - Gross motor function classification (GMFC-MLD)   *Neurological function:*   - Mean change (SD) from baseline in nerve conduction velocity (NCV) - Mean change (SD) from baseline in total score for brain magnetic resonance (MR) imaging (Loes score) and sub-scores (demyelination, atrophy and tigroid scores).   *Cognitive function*:   - Proportion (n/N; %) of individuals with cognitive impairment - Median (range) time to cognitive impairment - Mean change (SD) from baseline in neurocognitive function measured using the Intelligence Quotient (IQ) - Mean change (SD) from baseline in neurocognitive function measured using the Developmental Quotient (DQ) - Mean change (SD) from baseline in the Expressive Language Function Classification   Arylsulfatase (ARSA) activity:   - Change from baseline in ARSA activity in total peripheral blood mononuclear cells (PBMC) - Change from baseline in ARSA activity in leukocytes - Change from baseline in peripheral blood (PB) CD14+ cells - Change from baseline in cerebrospinal fluid (CSF)   *Health related quality of life (HRQoL):*   - Mean change (SD) from baseline in Caregiver Observed Metachromatic Leukodystrophy Functioning and Outcomes Reporting Tool (COMFORT) - Mean change (SD) from baseline in the (EQ-5D)   *Safety:*  Proportion (n/N; %) of patients experiencing the following safety outcomes (to include treatment related events, treatment emergent events, and all events, where separate data are available):   - Any adverse event - Serious adverse events - Fatal adverse events   Any specific event occurring in ≥ 5% of patients in any one study arm  *Economic:*   - Health-related quality of life - Utilities - Costs and use of resources - For economic evaluations: - Location of study - Summary of model and comparators - Patient population (key characteristics, average age) - Costs (intervention and comparator) - Patient outcomes (clinical outcomes, quality adjusted life expectancy (QALYs), life expectancy) - Results (annual cost savings, annual savings per patient, incremental cost per QALY (ICER)) |
| **Study type** | The following types of studies were included:   - RCTs - Prospective or retrospective single arm studies with > 5 participants - Prospective or retrospective cohort studies with > 5 participants - Any type of economic evaluation (cost-effectiveness analysis (CEA), cost only comparison, budget impact analysis (BIA) or cost of illness (COI) study   The following studies were excluded from the review:   - Studies ≤ 5 participants - Case reports - Cross-sectional studies |
| ***At the time of commissioning this review EJ and LJ patients were included. However, subsequently the treatment indication for OTL-200 has focused on LI and EJ patients. To preserve the integrity of the systematic review LJ patients and J patients (i.e. EJ and LJ) are still included, though the focus is on LI and EJ patients where possible.**  **Best supportive/symptomatic care included any of the following including combinations of any of the following: Management of dystonia, infections, seizures (if required) or secretions; pain relief/sedative drugs (if required); feeding support (including gastrostomy); psychological and social support (including specialist schooling); coordination of the multidisciplinary team and community care; genetic advice and planning; and end of life care  NOTE: Vector clone number (VCN) and % lentivirus (LV) + clone are outcomes only relevant for OTL-200 gene therapy, these were not recorded as key outcomes, but these data were added to comments section of any relevant study. | |

### Table 2: Summary of study designs

| **Study ID – NCT number**  **(sample size)** | **Design** | **Time period/duration** | **Location** | **Funding** |
| --- | --- | --- | --- | --- |
| **Bley 2013****^[5](#_ENREF_5)^ - NR**  **(n=9)*** | Retrospective; single arm | Treatment during period 1991 to 2011 | Europe (Germany) – 1 site - Universitätsklinik Hamburg Eppendorf, Hamburg, Germany | NR |
| **Bohringer 2010****^[6](#_ENREF_6)^ - NR**  **(n=8)*** | NR/unclear; single arm | NR | Europe (Germany) – 1 site - University Children's Hospital, Tübingen, Germany | NR |
| **Boucher 2015****^[7](#_ENREF_7)^ - NR**  **(n=31)** | Retrospective; single arm | Transplant between Jun 1984 & Apr 2013 | N. America (USA) – 1 site - University of Minnesota | Biostatistical support NIH grants P30 CA77598 and P01 CA6549 |
| **EUROCORD 2018****^[8](#_ENREF_8)^ - NR**  **(n=60 MLD)** | Retrospective; single arm | Performed between Sept 1996 & Aug 2013 | Worldwide~ – NR sites | Public/non-profit organisations including Sylvia Toth Charity Foundation; NIH; and NHLBI |
| **Groeschel 2016****^[9](#_ENREF_9)^* - NR**  **(n=65)** | Retrospective; comparative | 7.5 (range 3.0 to 19.7) yrs | Europe (Germany) – 3 sites - Berlin, Hamburg and Tübingen | German Federal Ministry of Education and Research (LEUKONET database); European Commission (European Leukotreat database) |
| **Kehrer 2014**[**^10^**](#_ENREF_10)*** - NR**  **(n=59)** | Retrospective and prospective; single arm | Recruitment during a 5yr period | Europe (Germany) – NR sites – LEUKONET database | German Federal Ministry of Education and Research |
| **LDM/1 study**[**^11^**](#_ENREF_11)**** - NR (n=41)** | Retrospective and prospective; single arm | Performed between 2000 & 2017 | Europe (Italy) – 1 site – the San Raffaele Scientific Institute, Milan, Italy | Fondazione Telethon, IRCCS San Raffaele Scientific Institute, GlaxoSmithKline (GSK) and Orchard Therapeutics |
| **Martin 2013****^[12](#_ENREF_12)^ - NR**  **(n=27)** | Retrospective; single arm | 5.1 (range: 2.4 to 14.7) yrs | N. America (USA) – 1 site - University of North Carolina, Chapel Hill's Program of Neurodevelopmental Function in Rare Disorders | The Trimper Children Foundation |
| **Orchard Study (OTL-200 arm****) - NCT01560182**  **(n=20) plus EAP (n=9)** [**^13^**](#_ENREF_13) | Prospective (Study 201222)^+^ with historical retrospective/prospective control (NHx natural history) | 8yrs | Europe (Italy) – 1 site - The Paediatric Clinical Research Unit at Ospedale San Raffaele, in Milan, Italy | FondazionevTelethon and OSR; GSK; Orchard Therapeutics |
| **Orchard Study Natural history arm) (n=31)**[**^13^**](#_ENREF_13) |  | NR |  |  |
| **Prasad 2008****^[14](#_ENREF_14)^ - NR**  **(n=15)** | Prospective; single arm | Recruitment & treatment between Aug 1995 & Apr 2007 | N. America (USA) – 1 site | Part funded by the NHLBI (Bethesda, MD) - NIH (grant no. N01-HB-67138) |
| **Singh 2012****^[15](#_ENREF_15)^ - NR**  **(n=11)** | Retrospective; single arm | NR/Unclear | Europe (UK) – 1 site | NR |
| **van Rappard 2016^[16](#_ENREF_16)^ - NR**  **(n=7)** | Retrospective; two arm comparative | Treatment between 2004 & 2015 | Europe (Netherlands) – 1 site | M.O. Knip Foundation |
| *** Indicates that there is a possibility of overlap with populations reported in other studies based in German study centres and/or using the LEUKONET database**  ****Indicated that there is a possibility of overlap of patients between two studies (i.e. LDM/1 study and the natural history arm of Orchard Study), however, the actual number is unclear.**  + non-randomised, open-label, prospective, comparative (non-concurrent control), single-centre phase I/II trial  ~ France, Germany; Italy; Spain; UK; Portugal; Poland; Australia; Canada; USA; Turkey; Israel; Slovakia; Netherlands; and Belgium  ^ Data cut-off date of 05 December 2018 and at this time median duration of follow-up in both studies combined was 1.495 years (range: 0.99 years to 2.72 years). Hospital Exemption Programme [HEP] - Study 205029 (2.5 to 2.7 years) and Compassionate use programme only [CUP] - Study 206258 (1.5 years or less) | | | | |
| *Abbreviations*: EAP Expanded Access Programme; GSK GlaxoSmithKline; NHLBI National Heart, Lung, and Blood Institute; NIH National Institutes of Health; NR not reported; OSR Ospedale San Raffaele; San Raffaele Telethon Institute for Gene Therapy (SR TIGET); yr year | | | | |

### Table 3: Summary of treatments in included studies

| **Study ID – NCT number**  **(sample size)** | **Treatment(s)** | **Supporting treatment(s)** |
| --- | --- | --- |
| **Bley 2013**[**^5^**](#_ENREF_5) **- NR**  **(n=9)*** | **HSCT (n=9):** No further details  **Conditioning:** NR | NR |
| **Bohringer 2010**[**^6^**](#_ENREF_6) **- NR**  **(n=8)*** | **HSCT (n=8):** n=6 received bone marrow from a 10/10 matched unrelated donor; n=1 from sibling; and n=1 with LI MLD from haploidentical mother. All received mean dose 7.74 × 10^6^ CD34+ cells/kg BW and engrafted at day 12 (range 11-22) with permanent full donor chimerism.  **Conditioning:** Treosulfan (3 × 14 g/m^2^), fludarabin (4 × 40 mg/m^2^) and thiotepa (10 mg/kg) | NR |
| **Boucher 2015**[**^7^**](#_ENREF_7) **- NR**  **(n=31)** | **HSCT (n=31):** n=4 MLD-carrier related BM donor (sibling) transplants; n=7 UCBT; n=3 double UCBT; n=6 related BMT donor; and n=11 unrelated BMT  **Conditioning:** Reduced-intensity conditioning (n=2) using Mel, clofarabine, low dose total-body irradiation and alemtuzumab. Myeloablative conditioning (n=29) with Bu/Cy n=29 (n=23 Bu/Cy; n=6 Cy/TBI) | GVHD prophylaxis, infectious disease prophylaxis, growth factor administration, and blood product support per University of Minnesota BMT Program standard of care guidelines. |
| **EUROCORD 2018**[**^8^**](#_ENREF_8) **- NR**  **(n=60 MLD)** | **HSCT (n=60):** Related or unrelated donor CBCT. All but one patient received unrelated donation  **Conditioning:** Majority MAC (97%) with most (83.4%) receiving Bu/Cy as part of regimen - NOTE: data are for the total population and not specifically children with MLD. | GVHD prophylaxis (cyclosporine-based regimen) in 97% of total population; 96% received anti-thymocyte globulin as serotherapy. |
| **Groeschel 2016**[**^9^**](#_ENREF_9)*** - NR**  **(n=65)** | **HSCT (n=65):** Allogeneic BMT (17/24 received this as only HSCT), cord blood or peripheral blood (4/27 received this as only HSCT) isolated from matched or mismatched family or non-family donors. n=3 received >1 HSCT (n=1 two PB HSCTs; n=1 CBCT then PB HSCT; and n=1 PB HSCT, then BM HSCT, then second BM HSCT).  **Conditioning:** Bu(12.8 mg/kg), Cy (120 mg/kg) with/without antithymoglobulin fresenius (30mg/kg) or thymoglobulin (10 mg/kg); Flu (150 mg/m²), treosulfan (42g/m²), thiotepa (10mg/kg) & thymoglobulin (10 mg/kg); further detail reported but not extracted.  **No treatment control (standard care) (n=41):** No information was reported on standard care or supporting treatments. | NR |
| **Kehrer 2014**[**^10^**](#_ENREF_10)***^ - NR**  **(n=59)** | **No treatment control (natural history) (n=59):** No information was reported on standard care or supporting treatments. | NR |
| **LDM/1 study**[**^11^**](#_ENREF_11)**** - NR (n=41)** | **No treatment control (natural history) (n=41):** No information was reported on standard care or supporting treatments. | NR |
| **Martin 2013**[**^12^**](#_ENREF_12) **- NR**  **(n=27)** | **HSCT (n=27):** Cord blood units with the highest nucleated cell dose, matching at least 4/6 HLA loci. Units with closest HLA match, highest nucleated cell dose, and normal ARSA activity were selected for use.  **Conditioning:** SMAC (n=1) with alemtuzumab (3.2 mg/kg), Flu (150 mg/m^2^), Mel (140 mg/m^2^), thiotepa (200 mg/m^2^), and hydroxyurea. MAC(n=26) with Bu (16 doses 20-40 mg/m^2^ per dose to target 600-900 ng/mL), Cy (200 mg/kg), and horse ATG (90 mg/kg) | GVHD prophylaxis (9mths cyclosporine and methylprednisolone/mycophenolic acid for 2-3mths if GVHD not active. Other supportive care administered (NR). |
| **Orchard Study 201222 (OTL-200 arm) NCT01560182 plus EAP**  **(n=29)** [**^13^**](#_ENREF_13) | **OTL-200 (Study 201222) (n=20):** CD34+ HSC and progenitor cells removed and genetically modified *ex-vivo* using lentiviral vector encoding for ARSA cDNA sequence under control of hPGK promoter. Cells suspended in saline (min 2 × 10^6^ CD34+ cells/kg, target 5 × 10^6^ to 10 × 10^6^ CD34+ cells/kg; max 20 × 10^6^ CD34+ cells/kg). After premedication (15-30 mins pre-administration) with chlorphenamine (0.25 mg/kg, max 10 mg) or equivalent (not corticosteroid), OTL-200 IV infused through CVC over 10 -20 mins), followed by saline wash. if needed, furosemide (0.2 to 1 mg/kg, max 20 mg) could be administered.  **Conditioning:** SMAC (n=13)- BW-based doses of IV Bu (< 9kg - 1mg/kg/dose; 9-<16kg - 1.2mg/kg/dose; 16-23kg- 1.1mg/kg/dose; >23-24kg - 0.95mg/kg/dose; >34kg 0.8mg/kg/dose). Total of 14 doses, as 2hr IV every 6 hrs Day -4 to Day -1. MAC (n=16) - Bu (16 doses 20-40 mg/m^2^ per dose - target 600-900 ng/mL), Cy (200 mg/kg), and horse ATG (90 mg/kg).  **No treatment control (natural history) (n=31):** Supportive care not explicitly described, however, in the text it is stated that ‘current therapies for symptomatic patients are generally supportive and aimed at managing disease complications and maintaining adequate quality of life, including physical therapy to maintain mobility, muscle relaxant medications to reduce spasticity, pain management, prevention of skeletal deformity, respiratory physiotherapy to manage pulmonary infections, anti-convulsant drugs to control seizures, and anti-psychotic medications to control psychiatric symptoms, as well as dietary support, enteral nutrition through a feeding tube in case of dysphagia, and family and psychological counselling’ | Multiple prior/concomitant medications typical for HSCT; prophylaxis pre, during and post-transplant included standard care antiemetics, analgesics, antiepileptics, oral decontamination medications, diuretics, and pre-medications for sedation/general anaesthesia. |
| **Natural history arm)** [**^13^**](#_ENREF_13)  **(n=31)** |  |  |
| **Prasad 2008**[**^14^**](#_ENREF_14) **- NR**  **(n=15)** | **HSCT (n=15):** Unrelated UBCT using units from 8 US public banks after matching on at least 3/6 HLA loci. Units with highest ARSA selected. Cells resuspended in dextran/albumin solution (max 5mg/kg of BW) and infused over 15-30min via CVC.  **Conditioning:** 16 doses of Bu oral or IV every 6hrs over 4 days (days 9 to 6) with PHT prophylaxis against seizures. Followed by Cy 50 mg/kg/dose for 4 days IV mesna prophylaxis against haemorrhagic cystitis (days 5 to 2), and equine ATG 30 mg/kg/dose IV QD for 3 days (days 3 to 1); adjusted to maintain a steady state 600 to 900 ng/mL. No radiation. | Treatments included (% in total population): chlorphenamine (25%); chlorphenamine maleate (25%); domperidone (25%); eculizumab (25%); gentamicin (25%); honey (25%); hydrocortisone (25%); lenograstim (25%); methylprednisolone (25%); metronidazole (25%); morphine (25%); piperacillin/ tazobactam (25%). |
| **Singh 2012**[**^15^**](#_ENREF_15) **- NR**  **(n=11)** | **Natural history (standard care) (n=11):** Not explicitly defined however, gastrostomy and advanced palliative care are mentioned as interventions which may impact mortality. | GVHD prophylaxis (yrs 1995-2004 - cyclosporine & methylprednisolone or after 2004 - cyclosporine & mycophenolate mofetil). Viral pathogens/Pneumocystis carinii prophylaxis (methylprednisolone or switch cyclosporine to tacrolimus plus daclizumab). Fungal infection prophylaxis (prior to 1999 - low-dose amphotericin-B, after 2000 – voriconazole). Broad spectrum antibiotic therapy for fever (IV Ig 500 mg/kg per dose/QW until day 100 then monthly until discontinuation of GVHD therapy or Ab production). Veno-occlusive disease prophylaxis (continuous IV 100 U/kg per day heparin day 10 to day 28) TPN transfusions of leukocyte-depleted & irradiated packed RBC and platelets, and GSF 10 µg/kg/QD IV from day 1 until WBC > 5 x 10^9^/L (5000/µL). |
| **van Rappard 2016**[**^16^**](#_ENREF_16) **- NR**  **(n=26)** | **HSCT (n=7):** Either HLA identical sibling (n=3; non-carrier) or an unrelated UCBT (n=10) (min match 4/6 HLA loci).  **Conditioning:** Bu (75 to 90 mg*h/L) and ATG (10 -2.5 mg/kg/day, day -8 till -4) plus either Cy (200 mg/kg total) or Flu (160 mg/m^2^ or 40 mg/m^2^ from day -5 till -1).  **Natural history (standard care) (n=19):** No details of standard care were reported however, intervention free survival (IFS) included death, wheelchair dependency, gastrostomy, and intrathecal baclofen treatment as events | All received cyclosporine (target 200–250 μg/L) as GVHD prophylaxis. Prednisone given to cord blood recipients from day 0 until day 28, 1 mg/kg per day, as well as methotrexate 10 mg/m2 at days 1, 3 & 6. |
| *** Indicates that there is a possibility of overlap with populations reported in other studies based in German study centres and/or using the LEUKONET database**  ****Indicated that there is a possibility of overlap of patients between two studies (i.e., LDM/1 study and the natural history arm of Orchard Study), however, the actual number is unclear.**  **^One additional paper was identified during the 2021 update which included more patients (LI: n=35; EJ: n=18; LJ: n=28) and longer follow-up (the actual length was not reported). The overlap between previously reported patients and the new publication is not clearly described in text, thus, the results are included in Appendix 13.** | | |
| *Abbreviations*: Ab antibody; ARSA arylsulfatase A; ATG antithymocyte globulin; BM bone marrow; BMT bone marrow transplants; Bu busulfan; BW body weight; CBCT cord blood cell transplant; CVC central venous catheter; Cy cyclophosphamide; cDNA complementary DNA; DNA deoxyribonucleic acid; Flu Flu; GSF granulocyte colony stimulating factor; GVHD graft versus host disease; HLA human leukocyte antigen; hr hour; HSC haematopoietic stem cells; HSCT haematopoietic stem cell transplant; Ig immunoglobulin; IV intravenous; MAC myeloablative conditioning; max maximum; min minute or minimum; NA not applicable; NR not reported; hPGK human phosphoglycerate kinase; PB peripheral blood; PHT phenytoin; QD daily/once a day; QW once a week; RBC red blood cells; SMAC sub-myeloablative conditioning; TBI total body irradiation; TPN total parenteral nutrition; UCBT umbilical cord blood transplants; WBC white blood cell count | | |

###

### Table 4: Change in gross motor function (GMFM-MLD score or GMFC-MLD) for standard care

| **Treatment** | **MLD type** | **Symptom status** | **Time point** | **Outcome definition** | **n/N (%)** | **Source** |
| --- | --- | --- | --- | --- | --- | --- |
| **Standard care** | LI | NR/unclear | Last available follow-for each patient^ | No. with worsening GMFM score | 2/6 (33.3%) | van Rappard 2016[^16^](#_ENREF_16) - R |
|  | LI | NR/unclear | Last available follow-for each patient^ | No. with worsening GMFM score | 4/6 (66.7%) | van Rappard 2016[^16^](#_ENREF_16) - R |
|  | J | NR/unclear | Last available follow-for each patient^ | No. with no change in GMFM score | 12/12 (100%) | van Rappard 2016[^16^](#_ENREF_16) - R |
|  | J | Mixed | 10yrs** | Progression to GMFC-MLD level 5^1^ | 28/41 (68.29%) | Groeschel 2016[^9^](#_ENREF_9) – R* |
| *** Indicates that there is a possibility of overlap with populations reported in other studies based in German study centres and/or using the LEUKONET database**  Time point is reported as described by the author(s), where possible the baseline from which time is measured is stated: ^After treatment; ** After disease onset  ^1^ GMFC-MLD Level 5 corresponds to ‘only head control possible’  Mixed refers to populations with a mixture of pre-symptomatic and symptomatic patients | | | | | | |
| *Abbreviations*: GMFM Gross Motor Function Measure; I infantile MLD; J Juvenile MLD; LI late infantile MLD; HSCT haemopoietic stem cell transplantation; MLD metachromatic leukodystrophy; mth month; No. number of patients; n number with outcome; N total number analysed; NR not reported; P prospective study; R retrospective study; SD stable disease; yr year | | | | | | |

### Table 5: Change in gross motor function (GMFC-MLD) for standard care

| **MLD type** | **Symptom status** | **Outcome definition** | **N analysed** | **Median time(IQR 25% to 75% or range)** | **Source** |
| --- | --- | --- | --- | --- | --- |
| LI | Mixed | Age at entry to Level 1 of GMFC-MLD | NR | Median 18mths (IQR 18 to 20) | Kehrer 2014[^17^](#_ENREF_17) |
| LI | Mixed | Age at entry to Level 2 of GMFC-MLD | NR | Median 18mths (IQR 18 to 26.5) | Kehrer 2014[^17^](#_ENREF_17) |
| LI | Mixed | Age at entry to Level 3 of GMFC-MLD | NR | Median 28mths (IQR 22.5 to 29.5) | Kehrer 2014[^17^](#_ENREF_17) |
| LI | Mixed | Age at entry to Level 4 of GMFC-MLD | NR | Median 29.5mths (IQR 26.5 to 31.3) | Kehrer 2014[^17^](#_ENREF_17) |
| LI | Mixed | Age at entry to Level 5 of GMFC-MLD | NR | Median 31mths (IQR 28.5 to 34) | Kehrer 2014[^17^](#_ENREF_17) |
| LI | Mixed | Age at entry to Level 6 of GMFC-MLD | NR | Median 33.5mths (IQR 31 to 36) | Kehrer 2014[^17^](#_ENREF_17) |
| J | Mixed | Age at entry to Level 1 of GMFC-MLD | NR | Median 64.5mths (IQR 47 to 92) | Kehrer 2014[^17^](#_ENREF_17) |
| J | Mixed | Age at entry to Level 2 of GMFC-MLD | NR | Median 91mths (IQR 72.3 to 114.8) | Kehrer 2014[^17^](#_ENREF_17) |
| J | Mixed | Age at entry to Level 5 of GMFC-MLD | NR | Median 96mths (IQR 78 to 157) | Kehrer 2014[^17^](#_ENREF_17) |
| J | Mixed | Age at entry to Level 6 of GMFC-MLD | NR | Median 116mths (IQR 84.8 to 159) | Kehrer 2014[^17^](#_ENREF_17) |
| LI | Mixed | Time interval from entry into Level 1 to Level 2 of GMFC-MLD | NR | Median 8mths (IQR 6 to 13) | Kehrer 2014[^17^](#_ENREF_17) |
| LI | Mixed | Time interval from entry into Level 2 to Level 5 of GMFC-MLD | NR | Median 12mths (IQR 5 to 16) | Kehrer 2014[^17^](#_ENREF_17) |
| LI | Mixed | Time interval from entry into Level 5 to Level 6 of GMFC-MLD | NR | Median 2mths (IQR 1 to 3) | Kehrer 2014[^17^](#_ENREF_17) |
| LI | Mixed | Time interval from entry into Level 1 to Level 6 of GMFC-MLD | NR | Median 15mths (IQR 10 to 18) | Kehrer 2014[^17^](#_ENREF_17) |
| J | Mixed | Time interval from entry into Level 1 to Level 2 of GMFC-MLD | NR | Median 27mths (IQR 18 to 52) | Kehrer 2014[^17^](#_ENREF_17) |
| J | Mixed | Time interval from entry into Level 2 to Level 5 of GMFC-MLD | NR | Median 5mths (IQR 3 to 22) | Kehrer 2014[^17^](#_ENREF_17) |
| J | Mixed | Time interval from entry into Level 5 to Level 6 of GMFC-MLD | NR | Median 12mths (IQR 4 to 27) | Kehrer 2014[^17^](#_ENREF_17) |
| J | Mixed | Time interval from entry into Level 1 to Level 6 of GMFC-MLD | NR | Median 75mths (IQR 41 to 94) | Kehrer 2014[^17^](#_ENREF_17) |
| EJ | NR/unclear | Time interval from entry into Level 1 to Level 2 of GMFC-MLD | 12/14 | Median 0.75 years (range 0.09 to 3.92) | LDM/1 study[^11^](#_ENREF_11)** |
| LJ | NR/unclear | Time interval from entry into Level 1 to Level 2 of GMFC-MLD | 1/5 | 1.02 years | LDM/1 study[^11^](#_ENREF_11)** |
| Summary of GMFC-MLD levels:  M0 Walking without support with quality and performance normal for age  M1 Walking without support but with reduced quality of performance (i.e. instability when standing or walking)  M2 Walking with support; walking without support not possible (fewer than five steps)  M3 Sitting without support AND locomotion such as crawling or rolling; walking with or without support not possible  M4 4a Sitting without support but no locomotion OR 4b sitting without support not possible, but locomotion such as crawling or rolling  M5 No locomotion nor sitting without support, but head control possible  M6 Loss of any locomotion as well as loss of any head and trunk control  Mixed refers to populations with a mixture of pre-symptomatic and symptomatic patients | | | | | |
| *Disease progression of LJ patients was further subcategorised by type of first symptoms at disease onset i.e. motor ± cognitive (motor: e.g. gain abnormalities, GMFC-MLD level 1, abnormal movement patterns, etc.; ±cognitive) and cognitive (e.g. concentration problems, decline in school performance, reduced working speed, etc.). Number of patients per subcategory was NR.  **The actual overlap of patients between LDM/1 study and the natural history arm of Orchard study is unclear.  *Abbreviations*: GMFM Gross Motor Function Measure-MLD; IQR interquartile range; J Juvenile MLD; LI late infantile MLD; MLD metachromatic leukodystrophy; mth month; N total number analysed; NR not reported; yrs years | | | | | |

### Table 6: Proportion of individuals showing cognitive impairment after HSCT or standard care

| **Treatment** | **MLD type** | **Symptom status** | **Time point** | **Outcome definition** | **n/N (&)** | **Source** |
| --- | --- | --- | --- | --- | --- | --- |
| **HSCT** | LI | Mixed | Post -HSCT* | No. with rapid cognitive decline in those patients exhibited cognitive impairment at baseline^+^ | 4/4 (100%) | Martin 2013[^12^](#_ENREF_12) - R |
|  | LI | Mixed | Post -HSCT* | No. continuing to have improved cognitive skills who did not show impairment at baseline^+^ | 4/5 (80%) | Martin 2013[^12^](#_ENREF_12) - R |
|  | LI | NR/unclear | Post -HSCT* | No. with cognitive deterioration~ | 1/2 (50%) | van Rappard 2016[^16^](#_ENREF_16) - R |
|  | LI | NR/unclear | Post -HSCT* | No. with cognitive function remaining the same~ | 1/2 (50%) | van Rappard 2016[^16^](#_ENREF_16) - R |
|  | J | NR/unclear | Post -HSCT* | No. with cognitive deterioration~ | 1/5 (20%) | van Rappard 2016[^16^](#_ENREF_16) - R |
|  | J | NR/unclear | Post -HSCT* | No. with cognitive function remaining the same ~ | 4/5 (80%) | van Rappard 2016[^16^](#_ENREF_16) - R |
| **Standard care** | LI to LJ** | NR/unclear | 22 to 72mths^ after diagnosis | No. with cognitive deterioration (not formally tested, clinically assessed) | 10/10 (100%) | van Rappard 2016[^16^](#_ENREF_16) - R |
| * exact time point NR  ** LJ disease is no longer of interest to the indication for OTL-200 treatment  ^+^ reported as measured using ‘standard tools’  ^ after diagnosis  ~ based on either Dutch versions of the Bayley Scales of Infant Development-II (BSID-II-NL; < 48 mths), the Wechsler Intelligence Scale for Children-III (WISC-III-NL; 6–18 yrs) or the Wechsler Nonverbal Scale of Ability (WNV-NL; 4–22 yrs).  Mixed refers to populations with a mixture of pre-symptomatic and symptomatic patients | | | | | | |
| *Abbreviations*: J juvenile MLD; LJ late juvenile MLD; HSCT haemopoietic stem cell transplantation; MLD metachromatic leukodystrophy; mth month; n number with outcome; N total number analysed; NR not reported; P prospective study; R retrospective study; yr year | | | | | | |

### Table 7: Age at decline in neurocognitive development in standard care (natural history) patients in Kehrer 2014^[10](#_ENREF_10)^

| **MLD type** | **Symptom status** | **Outcome definition** | **No. assessed** | **Median age/time in mths (IQR 25% and 75%)*** | **Source** |
| --- | --- | --- | --- | --- | --- |
| LI | Mixed | Age at onset of problems in concentration ^1^ | NR | Median NR (IQ 31 to NR) | Kehrer 2014[^10^](#_ENREF_10) |
| LI | Mixed | Age at onset of behavioural problems^2^ | NR | Median 31mths (IQ 28 to NR) | Kehrer 2014[^10^](#_ENREF_10) |
| LI | Mixed | Age at onset of decline in reading, writing, and calculating^3^ | NR | Median NR (IQR NR) | Kehrer 2014[^10^](#_ENREF_10) |
| LI | Mixed | Time after disease onset to cognitive decline for problems in concentration to appear^1^ | NR | Median NR (IQR 16 to NR) | Kehrer 2014[^10^](#_ENREF_10) |
| LI | Mixed | Time after disease onset to cognitive for behavioural problems to appear^2^ | NR | Median 18mths (IQR 9 to 21) | Kehrer 2014[^10^](#_ENREF_10) |
| LI | Mixed | Time after disease onset to cognitive decline for problems in reading, writing, and calculating to appear^3^ | NR | Median NR (IQR NR) | Kehrer 2014[^10^](#_ENREF_10) |
| J | Mixed | Age at onset of problems in concentration ^1^ | NR | Median 84mths (IQR 62 to 113) | Kehrer 2014[^10^](#_ENREF_10) |
| J | Mixed | Age at onset of behavioural problems^2^ | NR | Median 142mths (IQR 78 to 192) | Kehrer 2014[^10^](#_ENREF_10) |
| J | Mixed | Age at onset of decline in reading, writing, and calculating^3^ | NR | Median 128mths (IQR 87 to 180) | Kehrer 2014[^10^](#_ENREF_10) |
| J | Mixed | Time after disease onset to cognitive decline for problems in concentration to appear^1^ | NR | Median 6mths (IQR 0 to 14) | Kehrer 2014[^10^](#_ENREF_10) |
| J | Mixed | Time after disease onset to cognitive for behavioural problems to appear^2^ | NR | Median 32mths (IQR 6 to NR) | Kehrer 2014[^10^](#_ENREF_10) |
| J | Mixed | Time after disease onset to cognitive decline for problems in reading, writing, and calculating to appear^3^ | NR | Median 48mths (IQR 12 to 70) | Kehrer 2014[^10^](#_ENREF_10) |
| ^1^ Recorded by parents including any aspects of an attention disorder covering poor concentration, lack of endurance, diminished attention, or slower working speed  ^2^ Recorded by parents and included bad temper, moods, unsocial or aggressive behaviour  ^3^ Recorded by parents and including problems concerning at least one of the following: reading, writing, or calculatingMixed refers to populations with a mixture of pre-symptomatic and symptomatic patients  * Calculated using Kaplan Meier | | | | | |
| *Abbreviations*: IQR interquartile range; J juvenile MLD; LJ late juvenile MLD; MLD metachromatic leukodystrophy; mth month; NR not reported; P prospective study; | | | | | |

### Table 8: Number of patients with worsening, improving and stable ELFC-MLD scores after HSCT reported in Boucher 2015^[7](#_ENREF_7)^

| **MLD type** | **Symptom status** | **Outcome definition** | **n/N (%)** |  |
| --- | --- | --- | --- | --- |
| LI | Mixed | No. showing worsening ELFC-MLD score from time of HSCT to last follow-up point | 1/1 (100%) |  |
| LI to J | Mixed | No. showing worsening ELFC-MLD score from time of HSCT to last follow-up point | 10/15 (66.7%) |  |
| J | Mixed | No. showing worsening ELFC-MLD score from time of HSCT to last follow-up point | 9/14 (64.3%) |  |
| LI | Mixed | No. showing a stable ELFC-MLD score from time of HSCT to last follow-up point | 0/1 (0%) |  |
| LI to J | Mixed | No. showing a stable ELFC-MLD score from time of HSCT to last follow-up point^ | 5/15 (33.3%) |  |
| J | Mixed | No. showing a stable ELFC-MLD score from time of HSCT to last follow-up point^ | 5/14 (35.7%) |  |
| LI | Mixed | No. showing an improvement in ELFC-MLD score from time of HSCT to last follow-up point | 0/1 (0%) |  |
| LI to J | Mixed | No. showing an improvement in ELFC-MLD score from time of HSCT to last follow-up point | 0/15 (0%) |  |
| J | Mixed | No. showing an improvement in ELFC-MLD score from time of HSCT to last follow-up point | 0/14 (0%) |  |
| Mixed refers to populations with a mixture of pre-symptomatic and symptomatic patients  * ELFC-MLD scores were only presented in a figure and displayed as individual patient data. In addition, no narrative summary of the data was presented in the paper. The numbers reported in this table have been counted from the figures and are not directly reported in the paper.  ^ Includes n=3 juvenile patients (all symptomatic at HSCT) in E0; n=1 juvenile (symptomatic at HSCT) in E4 and n=1 juvenile (symptomatic at HSCT) in E3  *ELFC-MLD stages*:  E0 Communicates in complete sentences at a quality and performance normal for age  E1 Communicates in complete sentences with reduced quality of performance for age  E2 Cannot communicate complete sentences, but able to use 2-word phrases  E3 Cannot communicate 2-word phrases, but able to use single meaningful works/ideas  E4 Complete loss of expressive language | | | | |
| *Abbreviations*: J juvenile MLD; LJ late juvenile MLD; MLD metachromatic leukodystrophy; n number with outcome; N total number assessed | | | | |

### Table 9: Age at decline in language in standard care (natural history) patients in Kehrer 2014^[10](#_ENREF_10)^

| **MLD type** | **Symptom status** | **Outcome definition** | **No. assessed** | **Median age/time in mths (IQR 25% and 75%)*** | **Notes** |
| --- | --- | --- | --- | --- | --- |
| LI | Mixed | Age at loss of complete sentences | NR | Median 30 (IQR 28 to 31) | 11 (48%) had never learned to speak in complete sentences. For those who reached a certain ability, language acquisition was within the normal age range for healthy term born children |
| LI | Mixed | Age at loss of two-word sentences | NR | Median 32 (IQR 30 to 36) | 4 (17%) had never learned to speak in complete sentences. For those who reached a certain ability, language acquisition was within the normal age range for healthy term born children |
| LI | Mixed | Age at loss of single meaningful words | NR | Median 32 (IQR 31 to 36) | All had acquired single meaningful words. For those who reached a certain ability, language acquisition was within the normal age range for healthy term born children |
| LI | Mixed | Age at first language decline | NR | Median 30 (IQR 26 to 31) | NR |
| LI | Mixed | Age at complete loss of expressive language | NR | Median 32 (IQR 28 to 36) | NR |
| LI | Mixed | Age at loss of any communication^1^ | NR | Median 51 (IQR 48 to NR) | NR |
| LI | Mixed | Time after disease onset to cognitive decline for loss of complete sentences | NR | Median 17 (IQR 11 to 19) | 11 (48%) had never learned to speak in complete sentences. For those who reached a certain ability, language acquisition was within the normal age range for healthy term born children |
| LI | Mixed | Time after disease onset to cognitive decline for loss of two-word sentences | NR | Median 17 (IQR 12 to 20) | 4 (17%) had never learned to speak in complete sentences. For those who reached a certain ability, language acquisition was within the normal age range for healthy term born children |
| LI | Mixed | Time after disease onset to cognitive decline for loss of single meaningful words | NR | Median 17 (IQR 11 to 20) | All had acquired single meaningful words. For those who reached a certain ability, language acquisition was within the normal age range for healthy term born children |
| LI | Mixed | Time after disease onset to cognitive decline for first language decline | NR | Median 12 (IQR 9 to 16) | NR |
| LI | Mixed | Time after disease onset to cognitive decline for complete loss of expressive language | NR | Median 15 (IQR 12 to 20) | NR |
| LI | Mixed | Time after disease onset to cognitive decline for loss of any communication | NR | Median 33 (IQR 30 to NR) | NR |
| J | Mixed | Age at loss of complete sentences | NR | Median 132 (IQR 84 to NR) | All had learnt to speak in complete sentences |
| J | Mixed | Age at loss of two-word sentences | NR | Median 174 (IQR 90 to NR) |  |
| J | Mixed | Age at loss of single meaningful words | NR | Median NR (IQR 98 to NR) |  |
| J | Mixed | Age at first language decline | NR | Median 98 (IQR 69 to 192) |  |
| J | Mixed | Age at complete loss of expressive language | NR | Median 153 (IQR 87 to NR) |  |
| J | Mixed | Age at loss of any communication^1^ | NR | Median NR (IQR 192 to NR) |  |
| J | Mixed | Time after disease onset to cognitive decline for loss of complete sentences | NR | Median 72 (IQR 12 to NR) |  |
| J | Mixed | Time after disease onset to cognitive decline for loss of two-word sentences | NR | Median 96 (IQR 38 to NR) |  |
| J | Mixed | Time after disease onset to cognitive decline for loss of single meaningful words | NR | Median NR (IQR 43 to NR) |  |
| J | Mixed | Time after disease onset to cognitive decline for first language decline | NR | Median 24 (IQR 5 to 72) |  |
| J | Mixed | Time after disease onset to cognitive decline for complete loss of expressive language | NR | Median 72 (IQR 16 to NR) |  |
| J | Mixed | Time after disease onset to cognitive decline for loss of any communication^2^ | NR | Median NR (IQR NR) |  |
| ^1^ Recorded by parents and included complete loss of verbal communication combined with loss of directed voluntary movements, reaction towards optic/acoustic stimuli as well as loss of the ability to fix and follow with the eyes  ^2^ Recorded by parents and included complete loss of verbal communication combined with loss of directed voluntary movements, reaction towards optic/acoustic stimuli as well as loss of the ability to fix and follow with the eyes  Mixed refers to populations with a mixture of pre-symptomatic and symptomatic patients | | | | | |
| *Abbreviations*: IQR interquartile range; J juvenile MLD; LJ late juvenile MLD; MLD metachromatic leukodystrophy; mth month; NR not reported; P prospective study; R retrospective study; yr year | | | | | |

### Table 10: Number of patients with different MRI outcomes after HSCT

| **MLD type** | **Symptom status** | **Time point (mths)** | **Outcome definition** | **n/N (%)** | **Source** |
| --- | --- | --- | --- | --- | --- |
| LI | Mixed | 1yr post HSCT | No. with ‘MRI better than previous time point’ in demyelination on MRI^ | 2/3 (66.7%) | Boucher 2015[^7^](#_ENREF_7) - R |
| LI | Mixed | 2yrs post HSCT | No. with ‘MRI better than previous time point’ in demyelination on MRI^ | 1/2 (50%) | Boucher 2015[^7^](#_ENREF_7) - R |
| LI | Mixed | 1yr post HSCT | No. with ‘MRI same as previous timepoint or as baseline’ in demyelination on MRI^ | 1/3 (33.3%) | Boucher 2015[^7^](#_ENREF_7) - R |
| LI | Mixed | 2yrs post HSCT | No. with ‘MRI same as previous timepoint or as baseline’ in demyelination on MRI^ | 0/2 (0%) | Boucher 2015[^7^](#_ENREF_7) - R |
| LI | Mixed | 1yr post HSCT | No. with ‘MRI worse than previous timepoint’ in demyelination on MRI^ | 0/3 (0%) | Boucher 2015[^7^](#_ENREF_7) - R |
| LI | Mixed | 2yrs post HSCT | No. with ‘MRI worse than previous timepoint’ in demyelination on MRI^ | 1/2 (50%) | Boucher 2015[^7^](#_ENREF_7) - R |
| LI to J | Mixed | 1yr post HSCT | No. with ‘MRI better than previous time point’ in demyelination on MRI^ | 2/11 (18.2%) * | Boucher 2015[^7^](#_ENREF_7) - R |
| LI to J | Mixed | 2yrs post HSCT | No. with ‘MRI better than previous time point’ in demyelination on MRI^ | 1/9 (11.1%) | Boucher 2015[^7^](#_ENREF_7) - R |
| LI to J | Mixed | 1yr post HSCT | No. with ‘MRI same as previous timepoint or as baseline’ in demyelination on MRI^ | 6/11 (54.5%) | Boucher 2015[^7^](#_ENREF_7) - R |
| LI to J | Mixed | 2yrs post HSCT | No. with ‘MRI same as previous timepoint or as baseline’ in demyelination on MRI^ | 7/9 (77.8%) | Boucher 2015[^7^](#_ENREF_7) - R |
| LI to J | Mixed | 1yr post HSCT | No. with ‘MRI worse than previous timepoint’ in demyelination on MRI^ | 3/11 (27.3%) | Boucher 2015[^7^](#_ENREF_7) - R |
| LI to J | Mixed | 2yrs post HSCT | No. with ‘MRI worse than previous timepoint’ in demyelination on MRI^ | 1/9 (11.1%) | Boucher 2015[^7^](#_ENREF_7) - R |
| J | Mixed | 1yr post HSCT | No. with ‘MRI better than previous time point’ in demyelination on MRI^ | 0/8 (0%) | Boucher 2015[^7^](#_ENREF_7) - R |
| J | Mixed | 2yrs post HSCT | No. with ‘MRI better than previous time point’ in demyelination on MRI^ | 0/7 (0%) | Boucher 2015[^7^](#_ENREF_7) - R |
| J | Mixed | 1yr post HSCT | No. with ‘MRI same as previous timepoint or as baseline’ in demyelination on MRI^ | 5/8 (71.4%) | Boucher 2015[^7^](#_ENREF_7) - R |
| J | Mixed | 2yrs post HSCT | No. with ‘MRI same as previous timepoint or as baseline’ in demyelination on MRI^ | 7/7 (100%) | Boucher 2015[^7^](#_ENREF_7) - R |
| J | Mixed | 1yr post HSCT | No. with ‘MRI worse than previous timepoint’ in demyelination on MRI^ | 3/8 (37.5%) | Boucher 2015[^7^](#_ENREF_7) - R |
| J | Mixed | 2yrs post HSCT | No. with ‘MRI worse than previous timepoint’ in demyelination on MRI^ | 0/7 (0%) | Boucher 2015[^7^](#_ENREF_7) - R |
| LI to LJ** | Mixed | Median 5.1 yrs (range 2.4 to 14.7) | Improvement in modified Loes score~ | 16/19 (84.2%) | Martin 2013[^12^](#_ENREF_12) - R |
| * Of evaluable patients at pre-transplant baseline 97% had an abnormal MRI  ** LJ disease is no longer of interest to the indication for OTL-200 treatment  ^ characterised according to the presence or absence of pathologic white matter findings and whether changes relative to the previous scan (worse, stable, or better) were noted by the interpreting neuroradiologist. Baseline was time of HSCT. Data were only reported in a figure which has been used to calculate these data.  ~ Atrophy was uncommon in the patient population pre-HSCT but was an expected treatment outcome; therefore, atrophy scores were not included in the total score (modified Loes score). The MRI assessor was aware of patient age, which was needed to assess the degree of myelination, but no other clinical information. Images of all patients except one were reviewed on a picture archiving and communication system workstation, which allowed windowing of images to optimise signal intensity of structures for scoring and direct correlation of structures in different imaging planes.  Mixed refers to populations with a mixture of pre-symptomatic and symptomatic patients | | | | | |
| *Abbreviations*: J Juvenile MLD; LI late infantile MLD; HSCT haemopoietic stem cell transplantation; MLD metachromatic leukodystrophy; No. number of patients; n number with outcome; N total number analysed; NR not reported; P prospective study; R retrospective study; yr year | | | | | |

## APPENDIX 1: SEARCH STRATEGIES

**2021 Update searches**

Where possible, all searches used in the original 2020 systematic review were re-run in their entirety to update this work. The appendices contain the original 2020 strategy for NHS EED. This search was not rerun, as this has been an archival resource since 2015. At the time of searching the original review was unable to include a search of the WHO ICTRP Trials database, as the resource was no longer accessible to non-WHO searchers due to COVID emergency access restrictions. This was reinstated for the 2021 update.

| **Database** | **Dates covered** | **Hits** |
| --- | --- | --- |
| Medline & In-Process Citations | 1946-2021/07/07 | 4102 |
| Medline Daily Update & ePubs Ahead-of-Print | up to 2021/07/07 | 14 |
| PubMed | up to 2021/07/08 | 142 |
| Embase | 1974-2021/07/07 | 2924 |
| CENTRAL | up to 2021/07/Iss7 | 185 |
| Science Citation Index (SCI) | 1988-2021/07/12 | 2162 |
| Northern Lights | 2010-2021/wk26 | 114 |
| NHS EED | up to 2015/03/31 | 1 |
| EconLit | up to 2021/07/12 | 2 |
| ClinTrials.gov | up to 2021/07/12 | 62 |
| WHO ICTRP | up to 2021/07/13 | 25 |
| Orphanet | up to 2021/07/13 | 16 |
| WORLDSymposium | 2018-2021 | 60 |
| **Total** |  | **9809*** |

*Includes searches run in 2020 for NHS EED and 2018-2020 results for WORLDSymposium. The total for only those searches run in 2021 is 9762

**Medline & In-Process Citations (Ovid): 1946-2021/07/07**

**Searched 8.7.21**

1 Leukodystrophy, Metachromatic/ (1251)

2 (MLD and (gene$ or ARSA or ASA or arylsulfatase or arylsulphatase or leukodystroph$ or leucodystroph$)).ti,ab,ot. (919)

3 (Metachromatic adj2 (leukoencephal$ or leucoencephal$ or leukodystroph$ or leucodystroph$)).ti,ab,ot,kw,kf,hw. (1725)

4 (("Arylsulfatase A" or "arylsulphatase A" or "epididymis secretory sperm binding protein") adj2 deficien$).ti,ab,ot,kw,kf,hw. (239)

5 Greenfield$ Disease.ti,ab,ot,kw,kf,hw. (6)

6 (Cerebroside adj2 (Sulfatase or Sulphatase) adj2 Deficien$).ti,ab,ot,kw,kf,hw. (7)

7 (cerebroside adj2 (sulfate or sulphate) adj2 storage disease).ti,ab,ot,kw,kf,hw. (0)

8 ((ASA or ESSPB or ARSA) adj2 Deficien$).ti,ab,ot,kw,kf,hw. (130)

9 Cerebroside Deficien$.ti,ab,ot,kw,kf,hw. (0)

10 ((diffuse or metachromatic) adj3 (Cerebral or brain) adj3 sclerosis).ti,ab,ot,kw,kf,hw. (2375)

11 ((sulfatide or sulphatide) adj2 lipidosis).ti,ab,ot,kw,kf,hw. (18)

12 (mckusick-25010 or mckusick25010).ti,ab,ot,kw,kf,hw. (0)

13 (sulfatidosis or sulphatidosis).ti,ab,ot,kw,kf. (18)

14 or/1-13 (4381)

15 animals/ not (animals/ and humans/) (4821500)

**16 14 not 15 (4102)**

**Medline Daily Update & ePubs Ahead-of-Print (Ovid): up to 2021/07/07**

**Searched 8.7.21**

1 Leukodystrophy, Metachromatic/ (0)

2 (MLD and (gene$ or ARSA or ASA or arylsulfatase or arylsulphatase or leukodystroph$ or leucodystroph$)).ti,ab,ot. (9)

3 (Metachromatic adj2 (leukoencephal$ or leucoencephal$ or leukodystroph$ or leucodystroph$)).ti,ab,ot,kw,kf,hw. (9)

4 (("Arylsulfatase A" or "arylsulphatase A" or "epididymis secretory sperm binding protein") adj2 deficien$).ti,ab,ot,kw,kf,hw. (2)

5 Greenfield$ Disease.ti,ab,ot,kw,kf,hw. (0)

6 (Cerebroside adj2 (Sulfatase or Sulphatase) adj2 Deficien$).ti,ab,ot,kw,kf,hw. (0)

7 (cerebroside adj2 (sulfate or sulphate) adj2 storage disease).ti,ab,ot,kw,kf,hw. (0)

8 ((ASA or ESSPB or ARSA) adj2 Deficien$).ti,ab,ot,kw,kf,hw. (2)

9 Cerebroside Deficien$.ti,ab,ot,kw,kf,hw. (0)

10 ((diffuse or metachromatic) adj3 (Cerebral or brain) adj3 sclerosis).ti,ab,ot,kw,kf,hw. (0)

11 ((sulfatide or sulphatide) adj2 lipidosis).ti,ab,ot,kw,kf,hw. (0)

12 (mckusick-25010 or mckusick25010).ti,ab,ot,kw,kf,hw. (0)

13 (sulfatidosis or sulphatidosis).ti,ab,ot,kw,kf. (0)

14 or/1-13 (14)

15 animals/ not (animals/ and humans/) (2544)

**16 14 not 15 (14)**

**PubMed (NLM) (Internet): up to 2021/07/08**

**Searched 8.7.21**

**#20 Search: (#18 AND #19) 142**

#19 Search: (((pubstatusaheadofprint[sb] OR publisher[sb] OR pubmednotmedline[sb])))

4,249,565

#18 Search: (#14 NOT #17) 2,123

#17 Search: (#15 NOT (#15 AND #16)) 3,473,929

#16 Search: human*[tiab] 2,913,768

#15 Search: ((rat[tiab] or rats[tiab] or mouse[tiab] or mice[tiab] or murine[tiab] or rodent[tiab] or rodents[tiab] or hamster[tiab] or hamsters[tiab] or pig[tiab] or pigs[tiab] or porcine[tiab] or rabbit[tiab] or rabbits[tiab] or animal[tiab] or animals[tiab] or dogs[tiab] or dog[tiab] or cats[tiab] or cow[tiab] or bovine[tiab] or sheep[tiab] or ovine[tiab] or monkey[tiab] or monkeys[tiab]))

4,331,166

#14 Search: #1 or #2 or #3 or #4 or #5 or #6 or #7 or #9 or #11 or #12 or #13 2,359

#13 Search: ((((("sulfatide lipidosis"[Title/Abstract]) OR "sulphatide lipidosis"[Title/Abstract]) OR "mckusick-25010"[Title/Abstract]) OR "mckusick25010"[Title/Abstract]) OR sulfatidosis[Title/Abstract]) OR sulphatidosis[Title/Abstract] 36

#12 Search: ((("diffuse Cerebral sclerosis"[Title/Abstract]) OR "metachromatic Cerebral sclerosis"[Title/Abstract]) OR "diffuse brain sclerosis"[Title/Abstract]) OR "metachromatic brain sclerosis"[Title/Abstract] 43

#11 Search: ((((("epididymis secretory sperm binding protein deficiencies"[Title/Abstract]) OR "epididymis secretory sperm binding protein deficienct"[Title/Abstract]) OR "Cerebroside Deficiency"[Title/Abstract]) OR "Cerebroside Deficiencies"[Title/Abstract]) OR "Cerebroside Deficient"[Title/Abstract]) 0

#9 Search: ((((("ESSBP Deficiency"[Title/Abstract]) OR "ESSBP Deficiencies"[Title/Abstract]) OR "ESSBP Deficient"[Title/Abstract]) OR "epididymis secretory sperm binding protein deficiency"[Title/Abstract]) OR "epididymis secretory sperm binding protein deficiencies"[Title/Abstract]) OR "epididymis secretory sperm binding protein deficient"[Title/Abstract] 0

#7 Search: ((((((("Cerebroside Sulphatase storage disease"[Title/Abstract]) OR "cerebroside Sulfatase storage disease"[Title/Abstract]) OR "ARSA Deficiency"[Title/Abstract]) OR "ARSA Deficiencies"[Title/Abstract]) OR "ARSA Deficient"[Title/Abstract]) OR "ASA Deficiency"[Title/Abstract]) OR "ASA Deficiencies"[Title/Abstract]) OR "ASA Deficienct"[Title/Abstract] 63

#6 Search: ("Cerebroside Sulfatase Deficiency"[Title/Abstract]) OR "Cerebroside Sulphatase Deficiency"[Title/Abstract] 1

#5 Search: ("Arylsulfatase A Deficiency"[Title/Abstract]) OR "Arylsulphatase A Deficiency"[Title/Abstract] 68

#4 Search: ("Greenfield Disease[Title/Abstract]) OR "Greenfields Disease[Title/Abstract]

4

#3 Search: ((("Metachromatic leukoencephalopathy"[Title/Abstract]) OR "Metachromatic leucoencephalopathy"[Title/Abstract]) OR "Metachromatic leukodystrophy"[Title/Abstract]) OR "Metachromatic leucodystrophy"[Title/Abstract] 1,383

#2 Search: ((MLD[Title/Abstract]) AND (gene*[Title/Abstract] OR ARSA[Title/Abstract] OR ASA[Title/Abstract] OR arylsulfatase[Title/Abstract] OR arylsulphatase[Title/Abstract] OR leukodystroph*[Title/Abstract] OR leucodystroph*[Title/Abstract])) 1,045

#1 Search: "Leukodystrophy, Metachromatic"[Mesh

**Embase (Ovid): 1974-2021/07/07**

**Searched 8.7.21**

1 Metachromatic leukodystrophy/ (2137)

2 (MLD and (gene$ or ARSA or ASA or arylsulfatase or arylsulphatase or leukodystroph$ or leucodystroph$)).ti,ab,ot. (1476)

3 (Metachromatic adj2 (leukoencephal$ or leucoencephal$ or leukodystroph$ or leucodystroph$)).ti,ab,ot,kw,hw. (2358)

4 (("Arylsulfatase A" or "arylsulphatase A" or "epididymis secretory sperm binding protein") adj2 deficien$).ti,ab,ot,kw,hw. (321)

5 Greenfield$ Disease.ti,ab,ot,kw,hw. (0)

6 (Cerebroside adj2 (Sulfatase or Sulphatase) adj2 Deficien$).ti,ab,ot,kw,hw. (45)

7 (cerebroside adj2 (sulfate or sulphate) adj2 storage disease).ti,ab,ot,kw,hw. (0)

8 ((ASA or ESSPB or ARSA) adj2 Deficien$).ti,ab,ot,kw,hw. (170)

9 Cerebroside Deficien$.ti,ab,ot,kw,hw. (0)

10 ((diffuse or metachromatic) adj3 (Cerebral or brain) adj3 sclerosis).ti,ab,ot,kw,hw. (35)

11 ((sulfatide or sulphatide) adj2 lipidosis).ti,ab,ot,kw,hw. (10)

12 (mckusick-25010 or mckusick25010).ti,ab,ot,kw,hw. (0)

13 (sulfatidosis or sulphatidosis).ti,ab,ot,kw. (18)

14 or/1-13 (3246)

15 animal/ (1515289)

16 animal experiment/ (2691055)

17 (rat or rats or mouse or mice or murine or rodent or rodents or hamster or hamsters or pig or pigs or porcine or rabbit or rabbits or animal or animals or dogs or dog or cats or cow or bovine or sheep or ovine or monkey or monkeys).ti,ab,ot,hw. (7014819)

18 or/15-17 (7014819)

19 exp human/ (22461462)

20 human experiment/ (549308)

21 or/19-20 (22463344)

22 18 not (18 and 21) (5340409)

**23 14 not 22 (2924)**

**Cochrane Central Register of Controlled Trials (CENTRAL) (Wiley):** **up to 2021/07/Iss7**

**Searched 8.7.21**

#1 MeSH descriptor: [Leukodystrophy, Metachromatic] this term only 5

#2 (MLD and (gene* or ARSA or ASA or arylsulfatase or arylsulphatase or leukodystroph* or leucodystroph*)):ti,ab,kw 82

#3 (Metachromatic near/2 (leukoencephal* or leucoencephal* or leukodystroph* or leucodystroph*)):ti,ab,kw 12

#4 (("Arylsulfatase A" or "Arylsulphatase A" or "epididymis secretory sperm binding protein") near/2 Deficien*):ti,ab,kw 2

#5 Greenfield* Disease:ti,ab,kw 106

#6 (Cerebroside near/2 (Sulfatase or Sulphatase) near/2 Deficien*):ti,ab,kw 1

#7 (cerebroside near/2 (sulfate or sulphate) near/2 storage disease):ti,ab,kw 0

#8 ((ARSA or ASA or ESSBP) near/1 Deficien*):ti,ab,kw 1

#9 Cerebroside Deficien*:ti,ab,kw 3

#10 ((diffuse or metachromatic) near/3 (Cerebral or brain) near/2 sclerosis):ti,ab,kw 7

#11 ((sulfatide or sulphatide) near/2 lipidosis):ti,ab,kw 0

#12 (mckusick-25010 or mckusick25010):ti,ab,kw 0

#13 (sulfatidosis or sulphatidosis):ti,ab,kw 0

**#14 #1 or #2 or #3 or #4 or #5 or #6 or #7 or #8 or #9 or #10 or #11 or #12 or #13 in Trials 185**

**Science Citation Index (Web of Science**): **1988-2021/07/12**

**Date searched: 12.7.21**

**#17 #13 not #16 2,162**

#16 #14 not (#14 and #15) 3,630,760

#15 TS=(human) 3,903,500

#14 TS=(rat or rats or mouse or mice or murine or rodent or rodents or hamster or hamsters or pig or pigs or porcine or rabbit or rabbits or animal or animals or dogs or dog or cats or cow or bovine or sheep or ovine or monkey or monkeys) 4,659,593

#13 #1 or #2 or #3 or #4 or #5 or #6 or #7 or #8 or #9 or #10 or #11 or #12 2,511

#12 TS=(sulfatidosis or sulphatidosis) 5

#11 TS=("mckusick-25010" or "mckusick25010") 0

#10 TS=((sulfatide or sulphatide) NEAR/2 lipidosis) 3

#9 TS=((diffuse or metachromatic) NEAR/3 (Cerebral or brain) NEAR/2 sclerosis) 10

#8 TS=(Cerebroside NEAR/1 Deficien*) 10

#7 TS=((ARSA or ASA or ESSBP) NEAR/1 Deficien*) 121

#6 TS=(cerebroside NEAR/2 (sulfate or sulphate) NEAR/2 storage disease) 15

#5 TS=(Cerebroside NEAR/2 (Sulfatase or Sulphatase) NEAR/2 Deficien*) 2

#4 TS=(Greenfield* NEAR/1 Disease) 0

#3 TS=(("Arylsulfatase A" or "Arylsulphatase A" or "epididymis secretory sperm binding protein") NEAR/2 Deficien*) 194

#2 TS=(Metachromatic NEAR/2 (leukoencephal* or leucoencephal* or leukodystroph* or leucodystroph*)) 1,426

#1 TS=(MLD and (gene* or ARSA or ASA or arylsulfatase or arylsulphatase or leukodystroph* or leucodystroph*)) 1,385

**Northern Light Life Sciences Conference Abstracts (Ovid): 2010-2021/wk26**

**Searched 8.7.21**

1 (MLD and (gene$ or ARSA or ASA or arylsulfatase or arylsulphatase or leukodystroph$ or leucodystroph$)).ti,ab. (81)

2 (Metachromatic adj2 (leukoencephal$ or leucoencephal$ or leukodystroph$ or leucodystroph$)).ti,ab,hw. (78)

3 (("Arylsulfatase A" or "arylsulphatase A" or "epididymis secretory sperm binding protein") adj2 deficien$).ti,ab,hw. (4)

4 Greenfield$ Disease.ti,ab,hw. (0)

5 (Cerebroside adj2 (Sulfatase or Sulphatase) adj2 Deficien$).ti,ab,hw. (0)

6 (cerebroside adj2 (sulfate or sulphate) adj2 storage disease).ti,ab,hw. (0)

7 ((ASA or ESSPB or ARSA) adj2 Deficien$).ti,ab,hw. (6)

8 Cerebroside Deficien$.ti,ab,hw. (0)

9 ((diffuse or metachromatic) adj3 (Cerebral or brain) adj3 sclerosis).ti,ab,hw. (4)

10 ((sulfatide or sulphatide) adj2 lipidosis).ti,ab,hw. (0)

11 (mckusick-25010 or mckusick25010).ti,ab,hw. (0)

12 (Sulfatidosis or sulphatidosis).ti,ab,hw. (0)

**13 or/1-12 (114)**

**NHS Economic Evaluation Database (NHS EED) (CRD): up to 2015/03/31**

**Searched 14.5.20**

1 MeSH DESCRIPTOR Leukodystrophy, Metachromatic IN NHSEED 0

2 (((MLD and (gene* or ARSA or ASA or arylsulfatase or arylsulphatase or leukodystroph* or leucodystroph*)))) and ((Economic evaluation:ZDT and Bibliographic:ZPS) OR (Economic evaluation:ZDT and Abstract:ZPS)) IN NHSEED 1

3 (((Metachromatic near2 (leukoencephal* or leucoencephal* or leukodystroph* or leucodystroph*)))) and ((Economic evaluation:ZDT and Bibliographic:ZPS) OR (Economic evaluation:ZDT and Abstract:ZPS)) IN NHSEED 0

4 (((("Arylsulfatase A" or "arylsulphatase A" or "epididymis secretory sperm binding protein") near2 deficien*))) and ((Economic evaluation:ZDT and Bibliographic:ZPS) OR (Economic evaluation:ZDT and Abstract:ZPS)) IN NHSEED 0

5 (("Greenfield Disease" or "Greenfield Disease")) and ((Economic evaluation:ZDT and Bibliographic:ZPS) OR (Economic evaluation:ZDT and Abstract:ZPS)) IN NHSEED 0

6 (((Cerebroside near2 (Sulfatase or Sulphatase) near2 Deficien*))) and ((Economic evaluation:ZDT and Bibliographic:ZPS) OR (Economic evaluation:ZDT and Abstract:ZPS)) IN NHSEED 0

7 (((cerebroside near2 (sulfate or sulphate) near2 "storage disease"))) and ((Economic evaluation:ZDT and Bibliographic:ZPS) OR (Economic evaluation:ZDT and Abstract:ZPS)) IN NHSEED 0

8 ((((ASA or ESSPB or ARSA) near2 Deficien*))) and ((Economic evaluation:ZDT and Bibliographic:ZPS) OR (Economic evaluation:ZDT and Abstract:ZPS)) IN NHSEED 0

9 ((("Cerebroside Deficiency" or "Cerebroside Deficiencies" or "Cerebroside Deficient"))) and ((Economic evaluation:ZDT and Bibliographic:ZPS) OR (Economic evaluation:ZDT and Abstract:ZPS)) IN NHSEED 0

10 ((((diffuse or metachromatic) near3 (Cerebral or brain) near3 sclerosis))) and ((Economic evaluation:ZDT and Bibliographic:ZPS) OR (Economic evaluation:ZDT and Abstract:ZPS)) IN NHSEED 0

11 ((((sulfatide or sulphatide) near2 lipidosis))) and ((Economic evaluation:ZDT and Bibliographic:ZPS) OR (Economic evaluation:ZDT and Abstract:ZPS)) IN NHSEED 0

12 (((mckusick-25010 or mckusick25010))) and ((Economic evaluation:ZDT and Bibliographic:ZPS) OR (Economic evaluation:ZDT and Abstract:ZPS)) IN NHSEED 0

13 (((sulfatidosis or sulphatidosis))) and ((Economic evaluation:ZDT and Bibliographic:ZPS) OR (Economic evaluation:ZDT and Abstract:ZPS)) IN NHSEED 0

**14 #1 OR #2 OR #3 OR #4 OR #5 OR #6 OR #7 OR #8 OR #9 OR #10 OR #11 OR #12 OR #13 1**

**EconLit (EBSCO): up to 2021/07/12**

**Searched 12.7.21**

**S7 S1 OR S2 OR S3 OR S4 OR S5 OR S6 2**

S6 TX ( (Cerebroside N2 (Sulfatase or Sulphatase) N2 Deficien*) ) OR TX ( (cerebroside N2 (sulfate or sulphate) N2 storage disease) ) OR TX ( ((ARSA or ASA or ESSBP) N1 Deficien*) ) 0

S5 TX ((mckusick-25010 or mckusick25010) ) OR TX ((sulfatidosis or sulphatidosis)) 0

S4 TX Cerebroside Deficien* OR TX ( ((diffuse or metachromatic) N3 (Cerebral or brain) N2 sclerosis) ) OR TX ( ((sulfatide or sulphatide) N2 lipidosis) ) 0

S3 TX ( (Cerebroside N2 (Sulfatase or Sulphatase) N2 Deficien*) ) OR TX ( (cerebroside N2 (sulfate or sulphate) N2 storage disease) ) OR TX ( ((ARSA or ASA or ESSBP) N1 Deficien*) ) 0

S2 TX ( (Metachromatic N2 (leukoencephal* or leucoencephal* or leukodystroph* or leucodystroph*)) ) OR TX ( (("Arylsulfatase A" or "Arylsulphatase A" or "epididymis secretory sperm binding protein") N2 Deficien*) ) OR TX Greenfield* Disease 0

S1 AB ( (MLD and (gene* or ARSA or ASA or arylsulfatase or arylsulphatase or leukodystroph* or leucodystroph*)) ) OR TI ( (MLD and (gene* or ARSA or ASA or arylsulfatase or arylsulphatase or leukodystroph* or leucodystroph*)) ) 2

**NIH Clinicaltrials.gov (Internet)** [**https://clinicaltrials.gov/**](https://clinicaltrials.gov/)**: up to 2021/07/12**

**Searched 12.7.21**

Expert search:

sulfatidosis OR sulphatidosis OR mckusick-25010 OR mckusick25010 OR "sulfatide lipidosis" OR "sulphatide lipidosis" OR "diffuse brain sclerosis" OR "metachromatic brain sclerosis" OR "diffuse Cerebral sclerosis" OR "metachromatic Cerebral sclerosis" OR "Cerebroside Deficiency" OR "Cerebroside Deficiencies" OR "Cerebroside Deficient" OR "ARSA Deficiency" OR "ARSA Deficiencies" or "ARSA Deficient" OR "ESSBP Deficiency" OR "ESSBP Deficiencies" or "ESSBP Deficient" OR "Cerebroside Sulphatase storage disease" OR "Cerebroside Sulfatase storage disease" OR "Cerebroside Sulfatase Deficiency" OR "Cerebroside Sulphatase Deficiency" OR "Arylsulfatase A Deficiency" OR "Greenfield Disease" OR "Greenfields Disease" OR "Metachromatic leukoencephalopathy" OR "Metachromatic leucoencephalopathy" OR "Metachromatic leukodystrophy" OR "Metachromatic leucodystrophy" OR (MLD AND (gene OR genes OR genetic OR ARSA OR ASA OR arylsulfatase OR arylsulphatase OR leukodystrophy OR leucodystrophy))

**n = 62**

**WHO International Clinical Trials Registry Platform (ICTRP) (Internet): (**[**https://ictrptest.azurewebsites.net/AdvSearch.aspx**](https://ictrptest.azurewebsites.net/AdvSearch.aspx)**): up to 2021/07/13**

**Searched 13.7.21**

**Advanced search**

| **Search term** | **Hits** |
| --- | --- |
| sulfatidosis OR sulphatidosis OR mckusick-25010 OR mckusick25010 OR "sulfatide lipidosis" OR "sulphatide lipidosis" OR "diffuse brain sclerosis" OR "metachromatic brain sclerosis" OR "diffuse Cerebral sclerosis" OR "metachromatic Cerebral sclerosis" OR "Cerebroside Deficiency" OR "Cerebroside Deficiencies" OR "Cerebroside Deficient" OR "ARSA Deficiency" OR "ARSA Deficiencies" or "ARSA Deficient" OR "ESSBP Deficiency" OR "ESSBP Deficiencies" or "ESSBP Deficient" OR "Cerebroside Sulphatase storage disease" OR "Cerebroside Sulfatase storage disease" OR "Cerebroside Sulfatase Deficiency" OR "Cerebroside Sulphatase Deficiency" OR "Arylsulfatase A Deficiency" OR "Greenfield Disease" OR "Greenfields Disease" OR "Metachromatic leukoencephalopathy" OR "Metachromatic leucoencephalopathy" OR "Metachromatic leukodystrophy" OR "Metachromatic leucodystrophy | 24 |
| (MLD AND (gene OR genes OR genetic OR ARSA OR ASA OR arylsulfatase OR arylsulphatase OR leukodystrophy OR leucodystrophy)) | 17 |
| **Total** | **41** |
| **Total without duplicates** | **25** |

**Orphanet (Internet)**

**(**[**https://www.orpha.net/consor/cgi-bin/ResearchTrials_ClinicalTrials.php?lng=EN**](https://www.orpha.net/consor/cgi-bin/ResearchTrials_ClinicalTrials.php?lng=EN)**): up to** **2021/07/13**

**Searched 13.7.2**

Search term: Metachromatic Leukodystrophy

**16 trial records.**

**WORLDSymposium (Internet): 2018-2021**

**Searched 13.7.21 & 4.6.20**

**WORLDSymposium 2021**

Poster Session Abstracts

<https://worldsymposia.org/wp-content/uploads/WORLDSymposium2021-Poster-List.pdf>

| **Search term** | **Hits** |
| --- | --- |
| Metachromatic | 11 |
| MLD | 1 |
| Sulfatidosis | 0 |
| Sulphatidosis | 0 |
| ESSBP | 0 |
| ARSA | 2 |
| Greenfield | 0 |
| Leukodystrophy | 12 |
| leucodystrophy | 0 |
| **Total** | **26** |
| **Total after dedupes** | **12** |

Program

<https://worldsymposia.org/wp-content/uploads/WORLDSymposium-2021-Program.pdf>

| **Search term** | **Hits** |
| --- | --- |
| Metachromatic | 2 |
| MLD | 0 |
| Sulfatidosis | 0 |
| Sulphatidosis | 0 |
| ESSBP | 0 |
| ARSA | 1 |
| Greenfield | 0 |
| Leukodystrophy | 2 |
| leucodystrophy | 0 |
| **Total** | **5** |
| **Total after dedupe** | **2** |

**Searched 4.6.20**

**WORLDSymposium 2020**

Poster Session Abstracts

<https://worldsymposia.org/wp-content/uploads/WORLDSymposium2020-Poster-List.pdf>

| **Search term** | **Hits** |
| --- | --- |
| Metachromatic | 5 |
| MLD | 1 |
| Sulfatidosis | 0 |
| Sulphatidosis | 0 |
| ESSBP | 0 |
| ARSA | 0 |
| Greenfield | 0 |
| Leukodystrophy | 6 |
| leucodystrophy | 0 |
| **Total** | **12** |

Program

<https://worldsymposia.org/wp-content/uploads/WORLDSymposium-2020-Program.pdf>

| **Search term** | **Hits** |
| --- | --- |
| Metachromatic | 4 |
| MLD | 2 |
| Sulfatidosis | 0 |
| Sulphatidosis | 0 |
| ESSBP | 0 |
| ARSA | 0 |
| Greenfield | 0 |
| Leukodystrophy | 4 |
| leucodystrophy | 0 |
| **Total** | **8** |

**WORLDSymposium 2019**

Poster Session Abstracts

<https://www.worldsymposia.org/wp-content/uploads/WORLDSymposium-2019-Poster-List.pdf>

| **Search term** | **Hits** |
| --- | --- |
| Metachromatic | 2 |
| MLD | 1 |
| Sulfatidosis | 0 |
| Sulfatidosis | 0 |
| ESSBP | 0 |
| ARSA | 0 |
| Greenfield | 0 |
| Leukodystrophy | 3 |
| leucodystrophy | 0 |
| **Total** | **6** |

Program

<https://worldsymposia.org/wp-content/uploads/WORLDSymposium-Program-2019.pdf>

| **Search term** | **Hits** |
| --- | --- |
| Metachromatic | 3 |
| MLD | 0 |
| Sulfatidosis | 0 |
| Sulfatidosis | 0 |
| ESSBP | 0 |
| ARSA | 0 |
| Greenfield | 0 |
| Leukodystrophy | 0 |
| leucodystrophy | 0 |
| **Total** | **3** |

**WORLDSymposium 2018**

Poster Session Abstracts

<https://www.worldsymposia.org/wp-content/uploads/WORLDSymposium-2018-Poster-List.pdf>

| **Search term** | **Hits** |
| --- | --- |
| Metachromatic | 5 |
| MLD | 0 |
| Sulfatidosis | 0 |
| Sulfatidosis | 0 |
| ESSBP | 0 |
| ARSA | 0 |
| Greenfield | 0 |
| Leukodystrophy | 7 |
| leucodystrophy | 0 |
| **Total** | **12** |

Program

<https://worldsymposia.org/wp-content/uploads/WORLDSymposium-Program-2018.pdf>

| **Search term** | **Hits** |
| --- | --- |
| Metachromatic | 1 |
| MLD | 1 |
| Sulfatidosis | 0 |
| Sulfatidosis | 0 |
| ESSBP | 0 |
| ARSA | 0 |
| Greenfield | 0 |
| Leukodystrophy | 3 |
| leucodystrophy | 0 |
| **Total** | **5** |

## APPENDIX 2: LIST OF EXCLUDED STUDIES

### Table 11: Overall numbers of excluded studies and reason(s) for exclusion

|  | **Reason for exclusion** | **Number of papers** |
| --- | --- | --- |
| **The 2020 original systematic review** | Not relevant population (i.e. not ≤ 17 years and/or not infantile or juvenile MLD or no separate data for relevant MLD subpopulation) | 27 |
|  | No relevant outcome (i.e. not one of the listed outcomes of interest) | 13 |
|  | No extractable data (i.e. ongoing study with no published results or data presented in graphical form only) | 28 |
|  | Not relevant study design (i.e. ≤ 5 patients; case report; cross sectional study; or other design not of interest to the review) | 46 |
|  | Duplicate (i.e. the same publication not previously identified as a duplicate during the Endnote de-duplication process) | 4 |
|  | **TOTAL** | **118** |
| **The 2021 update** | Not relevant population (i.e. not ≤ 17 years and/or not infantile or juvenile MLD or no separate data for relevant MLD subpopulation) | 1 |
|  | No relevant outcome (i.e. not one of the listed outcomes of interest) | 14 |
|  | No extractable data (i.e. ongoing study with no published results or data presented in graphical form only) | 3 |
|  | Not relevant study design (i.e. ≤ 5 patients; case report; cross sectional study; or other design not of interest to the review) | 0 |
|  | Duplicate (i.e. the same publication not previously identified as a duplicate during the Endnote de-duplication process) | 22 |
|  | **TOTAL** | **40** |

### Table 12: List of studies excluded due to ‘Not relevant population’ – 27 studies (the 2020 original systematic review) and one study (the 2021 update)

|  | **Endnote #** | **Citation** |
| --- | --- | --- |
| **The 2020 original systematic review** | 97 | Darba J, Marsa A. Current Status and Use of Resources of Lysosomal Storage Diseases: Analysis of a Spanish Claims Database. Endocr Metab Immune Disord Drug Targets 2020;20(2):263-270. |
|  | 137 | Egger M, Davey Smith G, Schneider M, Minder C. Bias in meta-analysis detected by a simple, graphical test. BMJ 1997;315(7109):629-34. |
|  | 176 | Bonkowsky JL, Wilkes J, Shyr DC. Scope and Burden of Non-Standard of Care Hematopoietic Stem Cell Transplantation in Pediatric Leukodystrophy Patients. J Child Neurol 2018;33(14):882-887. |
|  | 256 | Mallhi KK, Smith AR, DeFor TE, Lund TC, Orchard PJ, Miller WP. Allele-Level HLA Matching Impacts Key Outcomes Following Umbilical Cord Blood Transplantation for Inherited Metabolic Disorders. Biol Blood Marrow Transplant 2017;23(1):119-125. |
|  | 513 | Brimley CJ, Lopez J, van Haren K, Wilkes J, Sheng X, Nelson C, et al. National variation in costs and mortality for leukodystrophy patients in US children's hospitals. Pediatr Neurol 2013;49(3):156-162.e1. |
|  | 522 | Mitchell R, Nivison-Smith I, Anazodo A, Tiedemann K, Shaw PJ, Teague L, et al. Outcomes of haematopoietic stem cell transplantation for inherited metabolic disorders: a report from the Australian and New Zealand Children's Haematology Oncology Group and the Australasian Bone Marrow Transplant Recipient Registry. Pediatr Transplant 2013;17(6):582-8. |
|  | 551 | Nelson C, Mundorff MB, Korgenski EK, Brimley CJ, Srivastava R, Bonkowsky JL. Determinants of health care use in a population-based leukodystrophy cohort. J Pediatr 2013;162(3):624-628.e1. |
|  | 922 | Ringden O, Remberger M, Svahn BM, Barkholt L, Mattsson J, Aschan J, et al. Allogeneic hematopoietic stem cell transplantation for inherited disorders: experience in a single center. Transplantation 2006;81(5):718-25. |
|  | 927 | Martin PL, Carter SL, Kernan NA, Sahdev I, Wall D, Pietryga D, et al. Results of the cord blood transplantation study (COBLT): outcomes of unrelated donor umbilical cord blood transplantation in pediatric patients with lysosomal and peroxisomal storage diseases. Biol Blood Marrow Transplant 2006;12(2):184-94. |
|  | 1073 | Koc ON, Day J, Nieder M, Gerson SL, Lazarus HM, Krivit W. Allogeneic mesenchymal stem cell infusion for treatment of metachromatic leukodystrophy (MLD) and Hurler syndrome (MPS-IH). Bone Marrow Transplant 2002;30(4):215-22. |
|  | 4108 | Seitelberger F. Neuropathology and genetics of Pelizaeus-Merzbacher disease. Brain Pathol 1995;5(3):267-73. |
|  | 4148 | Bonkowsky J, Wilkes J, Shyr D. Scope and burden of non-standard of care hematopoietic stem cell transplantation in pediatric leukodystrophy patients. Presented at the 47th National Meeting of the Child Neurology Society (CNS 2018); US. Ann Neurol 2018;84(Suppl 22):S331-S332. |
|  | 4305 | Van Rappard DF, Boelens JJ, Pouwels PJW, Hollak CEM, Van Der Knaap MS, Wolf NI. The effectiveness of hematopoietic cell transplantation (HSCT) in metachromatic leukodystrophy (MLD): Promising results. Presented at the 11th European Paediatric Neurology Society Congress (EPNS 2015); Vienna: Austria. Europ J Paediatr Neurol 2015;19(SUPPL. 1):S1. |
|  | 4316 | Kirgizov K, Pristanskov E, Skvortsova Y, Pechatnikova N, Persiantseva M, Sidorova N, et al. Hematopoietic stem cell transplantation in patients with neurometabolic diseases: A single center study of unique transplants in Russia. Presented at the 41st Annual Meeting of the European Society for Blood and Marrow Transplantation (EBMT 2015); Istanbul: Turkey. Bone Marrow Transplant 2015;50(SUPPL. 1):S496-S497. |
|  | 4366 | Hol J, Ruggeri A, Rocha VG, Michel G, Ayas M, O'Brien T, et al. Outcomes of allogeneic cord blood transplantation for leukodystrophies; a joint study of eurocord and "inborn errors WP-EBMT". Presented at the 2014 BMT Tandem Meetings; Grapevine: USA. Biol Blood Marrow Transplant 2014;20(2 SUPPL. 1):S86-S87. |
|  | 4371 | Hol J, Ruggeri A, Rocha V, Michel G, Ayas M, O'Brien T, et al. Outcomes of allogeneic cord blood transplantation for leukodystrophies; a joint study of eurocord and "inborn errors wp-ebmt". Presented at the 40th Annual Meeting of the European Group for Blood and Marrow Transplantation; Milan: Italy. Bone Marrow Transplant 2014;49(SUPPL. 1):S37-S38. |
|  | 4422 | Janna H, Ruggeri A, Rocha V, Michel G, Ayas M, O'Brien T, et al. Outcomes of allogeneic cord blood transplantation for leukodystrophies; A joint study of eurocord and "Inborn Errors WP-EBMT". Presented at the 55th Annual Meeting of the American Society of Hematology (ASH 2013); New Orleans: US. Blood 2013;122(21). |
|  | 4473 | Mitchell R, Smith NI, Teidemann K, Mechinaud F, Shaw PJ, Teague L, et al. Outcomes of haematopoietic stem cell transplantation for inherited metabolic disorders: A report from ANZCHOG and ABMTRR. Presented at the 2012 BMT Tandem Meetings; San Diego: US. Biol Blood Marrow Transplant 2012;18(2 SUPPL. 2):S301. |
|  | 6124 | Newman SK, Rupar T. Viral vector therapy as a therapeutic option for peripheral nerve disease associated with metachromatic leukodystrophy. Eur J Hum Genet 2019;27:1134-1134. |
|  | 6157 | Newman SK. Lentiviral-Vector-Mediated Gene Therapy for Metachromatic Leukodystrophy Decreases Sulfatide Accumulation in the CNS. Molecular Therapy 2019;27(4):373-373. |
|  | 6615 | Artigalas O, Lagranha VL, Pereira MLS, Burin MG, Giugliani R, Marques CL, et al. Clinical and biochemical study of 29 Brazilian patients with metachromatic leukodystrophy. Int J Clin Pharmacol Ther 2010;48:S75-S75. |
|  | 6842 | Consiglio A, Dolcetta D, Follenzi A, Luca T, Marchesini S, Gritti A, et al. Lentiviral vectors for in vivo gene therapy of metachromatic leukodystrophy. J Neurochem 2003;87:5-5. |
|  | 6860 | Biffi A, De Palma M, Quattrini A, Visigalli I, Dolcetta D, Bordignon C, et al. Lentiviral vector-based ex vivo gene therapy approach for metachromatic (MLD) and globoid (GLD) leukodystrophies. Molecular Therapy 2003;7(5):S88-S88. |
|  | 6861 | Consiglio A, Follenzi A, Dolcetta D, Marchesini S, Orlacchio A, Bordignon C, et al. In vivo gene therapy of metachromatic leukodystrophy by lentiviral vectors. FASEB J 2003;17(4):A685-A685. |
|  | 6905 | Koc ON, Day J, Brown D, Andrews P, Peters C, Nieder M, et al. Results of a phase I clinical trial of allogeneic mesenchymal stem cell (MSC) transplantation in patients with Hurler disease and metachromatic leukodystrophy (MLD). Blood 2000;96(11):170A-170A. |
|  | 6906 | Singh H, Scigliano E, Isola L, Richards S, Sivak M, Fruchtman S. Metachromatic leukodystrophy (MLD) treated with allogeneic bone marrow transplantion (BMT). A newer application to a previously lethal disorder. Blood 2000;96(11):365B-365B. |
|  | 7296 | Masonic Cancer Center UoM. Allogeneic Bone Marrow Transplant for Inherited Metabolic Disorders. NCT01043640. In: ClinicalTrials.gov [Internet]. Bethesda (MD): National Library of Medicine (US). 2015 [accessed 19.5.20]. Available from: https://ClinicalTrials.gov/show/NCT01043640 |
| **The 2021 update** | 7549 | Schlotawa L, Adang L, Preiskorn J, Friede T, Gaertner J, Ahrens-Nicklas R. A natural disease history study and a meta-analysis of published cases improve clinical knowledge on multiple sulfatase deficiency. J Inherit Metab Dis 2019;42(Suppl 1):27-8. |

### Table 13: List of studies excluded due to ‘No relevant outcome’ – 13 papers (the 2020 original systematic review) and 14 studies (the 2021 update)

|  | **Endnote #** | **Citation** |
| --- | --- | --- |
| **The 2020 original systematic review** | 205 | van Rappard DF, Konigs M, Steenweg ME, Boelens JJ, Oosterlaan J, van der Knaap MS, et al. Diffusion tensor imaging in metachromatic leukodystrophy. J Neurol 2018;265(3):659-668. |
|  | 4288 | Calabria A, Spinozzi G, Brasca S, Benedicenti F, Tenderini E, Naldini L, et al. Genomic integration site analysis of 7 metachromatic leukodystrophy patients up to 48 months follow-up after lentiviral hematopoietic stem cell gene therapy. Presented at the ESGCT and FSGT Collaborative Congress; Helsinki: Finland. Hum Gene Ther 2015;26(10):A64. |
|  | 4441 | Groschel S, Dali CI, Clas P, Kehrer C, Wilke M, Krageloh-Mann I. Metachromatic Leukodystrophy-cerebral grey and white matter volume changes and clinical course. Presented at the 38th Annual Meeting of the Society of Neuropediatrics; Munster: Germany. Neuropediatrics 2012;43(2). |
|  | 4563 | Groschel S, Kehrer C, Dali CI, Wilke M, Grodd W, Krageloh-Mann I. Metachromatic Leukodystrophy: Spatial and temporal pattern of MRI changes and their association with motor deterioration. Presented at the 36th Annual Meeting of the Society of Neuropediatrics; Mannheim: Germany. Neuropediatrics 2010;41(2). |
|  | 6012 | Calabria A, Spinozzi G, Rancoita P, Benedicenti F, Cesana D, Acquati S, et al. Characterization of Hematopoietic System Reconstitution In Vivo In Metachromatic Leukodystrophy Gene Therapy Patients. In: American Society of Gene and Cell Therapy Annual Meeting 2019; 29-Apr-2019, 2019. Available from: American Society of Gene and Cell Therapy (ASGCT)  https://discovery.northernlight.com/document.php?datasource=PHE&docid=PE20190607000001980&context=WK%40northernlight.com  https://ovidsp.ovid.com/ovidweb.cgi?T=JS&CSC=Y&NEWS=N&PAGE=fulltext&D=dscv7&AN=PE20190607000001980 |
|  | 6019 | Calabria A, Spinozzi G, Merelli I, Beretta S, Rancoita P, Benedicenti F, et al. Molecular Characterization of Hematopoietic System Reconstitution In Metachromatic Leukodystrophy Patients Following Hematopoietic Stem Cell Gene Therapy. In: American Society of Gene and Cell Therapy Annual Meeting 2018; 16-May-2018, 2018. Available from: American Society of Gene and Cell Therapy (ASGCT)https://discovery.northernlight.com/document.php?datasource=PHE&docid=PE20180529200004790&context=WK%40northernlight.com  https://ovidsp.ovid.com/ovidweb.cgi?T=JS&CSC=Y&NEWS=N&PAGE=fulltext&D=dscv6&AN=PE20180529200004790 |
|  | 6316 | Calabria A, Spinozzi G, Merelli I, Beretta S, Rancoita P, Benedicenti F, et al. Molecular characterization of hematopoietic system reconstitution in 7 metachromatic leukodystrophy patients following hematopoietic stem cell gene therapy. Hum Gene Ther 2016;27(11):A115-A116. |
|  | 7281 | Assistance Publique - Hôpitaux de P, European Leukodystrophy A. Imaging Study of the White Matter Lesions in Children With Metachromatic Leucodystrophy. NCT01325025. In: ClinicalTrials.gov [Internet]. Bethesda (MD): National Library of Medicine (US). 2016 [accessed 19.5.20]. Available from: https://ClinicalTrials.gov/show/NCT01325025 |
|  | 7289 | National Center for Research R, University of California LA. Stem Cell Transplantation (SCT) for Genetic Diseases. NCT00004378. In: ClinicalTrials.gov [Internet]. Bethesda (MD): National Library of Medicine (US). [accessed 19.5.20]. Available from: https://ClinicalTrials.gov/show/NCT00004378 |
|  | 7292 | Masonic Cancer Center UoM. MT2013-31: Allo HCT for Metabolic Disorders and Severe Osteopetrosis. NCT02171104. In: ClinicalTrials.gov [Internet]. Bethesda (MD): National Library of Medicine (US). 2020 [accessed 19.5.20]. Available from: https://ClinicalTrials.gov/show/NCT02171104 |
|  | 7303 | Paul S, University of P. Reduced Intensity Conditioning for Non-Malignant Disorders Undergoing UCBT, BMT or PBSCT. NCT01962415. In: ClinicalTrials.gov [Internet]. Bethesda (MD): National Library of Medicine (US). 2020 [accessed 19.5.20]. Available from: https://ClinicalTrials.gov/show/NCT01962415 |
|  | 7306 | University of P. Longitudinal Study of Neurodegenerative Disorders. NCT03333200. In: ClinicalTrials.gov [Internet]. Bethesda (MD): National Library of Medicine (US). 2030 [accessed 19.5.20]. Available from: https://ClinicalTrials.gov/show/NCT03333200 |
|  | 7316 | Duke U. Reduced Intensity Conditioning for Umbilical Cord Blood Transplant in Pediatric Patients With Non-Malignant Disorders. NCT00744692. In: ClinicalTrials.gov [Internet]. Bethesda (MD): National Library of Medicine (US). 2012 [accessed 19.5.20]. Available from: https://ClinicalTrials.gov/show/NCT00744692 |
| **The 2021 update** | 7366 | Pekgul F, Eroglu-Ertugrul NG, Bekircan-Kurt CE, Erdem-Ozdamar S, Cetinkaya A, Tan E, et al. Comprehensive clinical, biochemical, radiological and genetic analysis of 28 Turkish cases with suspected metachromatic leukodystrophy and their relatives. Mol Genet Metab Rep 2020;25:100688. |
|  | 7381 | Ammann-Schnell L, Groeschel S, Kehrer C, Frolich S, Krageloh-Mann I. The impact of severe rare chronic neurological disease in childhood on the quality of life of families - a study on MLD and PCH2. Orphanet J Rare Dis 2021;16(1):211. |
|  | 7460 | Adang LA, Groeschel S, Patel A, Cross Z, Elgun S, Kehrer C, et al. Clinical presentation of metachromatic leukodystrophy. J Inherit Metab Dis 2019;126(2):S19-S20. |
|  | 7473 | Jones S, Davison J, Mooney P, Campbell L, Baldock L, Wallington M, et al. Demographic and clinical characteristics of patients with metachromatic leukodystrophy in the United Kingdom: Interim results from an observational real-world study. Mol Genet Metab 2021;132(2):S53. |
|  | 7501 | Lum SH, Will A, Bonney D, Hiwarkar P, Poulton K, Church H, et al. Haemopoietic cell transplantation is a safe, long-term disease-modifying therapy for transplant-permissive inherited metabolic disease in the modern era. Bone Marrow Transplant 2019;53:P446. |
|  | 7514 | Eichler F, Pang F, Howie K, Walz M, Wilds A, Calcagni C, et al. Initial signs and symptoms of metachromatic leukodystrophy: a caregiver perspective. Mol Genet Metab 2021;132(2):S36. |
|  | 7524 | Calbi V, Fumagalli F, De Mattia F, Fratini E, Ferrua F, Barzaghi F, et al. Lentiviral haematopoietic stem cell gene therapy for metachromatic leukodystrophy: results in nine patients treated with a cryopreserved formulation of OTL-200. Mol Genet Metab 2021;132(2):S21-S22. |
|  | 7574 | Pang F, Dean R, Jensen I, Olaye A, Miller B. PRO30 The cost-effectiveness of OTL-200 for the treatment of metachromatic leukodystrophy (MLD). Value Health 2021;24(Suppl 1):S203. |
|  | 7579 | Pang F, Campbell L, Howie K, Wilds A, Calcagni C, Walz M. Quality of life of patients with metachromatic leukodystrophy and their caregivers in the US, UK, Germany and France. Mol Genet Metab 2021;132(2):S81. |
|  | 7723 | Escolar M, Yoon I, Bascou N, Poe M. Long-term neurodevelopmental, neurophysiological, and neuroradiological outcomes of hematopoietic stem cell transplantation for treatment of late-infantile metachromatic leukodystrophy. Mol Genet Metab 2021;129(2):S164-S165. |
|  | 7915 | Krivit W, Shapiro E, Lockman L, Kennedy W, Dhuna A, Ringden O, et al. Recommendations for treatment of metachromatic leukodystrophy by bone-marrow transplantation based on a review of 7 patients who have been engrafted for at least 1 year. In: Hobbs JR, Riches PG, editors. Correction of certain genetic diseases by transplantation 1991. Ruislip, UK: The CoGENT Trust, 1992: 57-72. |
|  | 8119 | Shire, Takeda. Natural history study of children with metachromatic leukodystrophy. NCT01963650 In: ClinicalTrials.gov [Internet]. Bethesda (MD): National Library of Medicine (US). 2016 [accessed 12.7.21]. Available from: https://ClinicalTrials.gov/show/NCT01963650 |
|  | 8173 | Eichler F. Initial signs and symptoms of metachromatic leukodystrophy: a caregiver perspective[ePoster 65]. Presented at WORLDSymposium 2021; 8-12 Feb 2021; Virtual Scientific Meeting. Mol Genet Metab 2021;132(2):1 |
|  | 8174 | Pang F. Quality of life of patients with metachromatic leukodystrophy and their caregivers in the US, UK, Germany and France [ePoster 186]. Presented at WORLDSymposium 2021; 8-12 Feb 2021; Virtual Scientific Meeting. Mol Genet Metab 2021;132(2):1. |

### Table 14: List of studies excluded due to ‘No extractable data’ – 28 papers (the 2020 original systematic review) and three studies (the 2021 update)

|  | **Endnote #** | **Citation** |
| --- | --- | --- |
| **The 2020 original systematic review** | 46 | Orchard Therapeutics. A safety and efficacy study of cryopreserved OTL-200 for treatment of metachromatic leukodystrophy (MLD). NCT03392987. In: ClinicalTrials.gov [Internet]. Bethesda (MD): National Library of Medicine (US). 2018 [accessed 19.11.19]. Available from: https://clinicaltrials.gov/ct2/show/NCT03392987?term=NCT03392987&rank=1 |
|  | 64 | Orchard Therapeutics. OTL-200 205756: a single arm, open label, clinical study of cryopreserved autologous CD34+ cells transduced with lentiviral vector containing human ARSA cDNA OTL-200, for the treatment of early onset metachromatic leukodystrophy (MLD). Abbreviated clinical study report (205756/v1.0)[PDF provided by Orchard]: Orchard Therapeutics, 4th October 2019 [accessed 14.4.20]. 92p. |
|  | 65 | Orchard Therapeutics. Clinical study report (205756/v1.0). 14: tables and figures [PDF provided by Orchard]: Orchard Therapeutics, 4th October 2019 [accessed 14.4.20]. 52p. |
|  | 121 | Elgun S, Waibel J, Kehrer C, van Rappard D, Bohringer J, Beck-Wodl S, et al. Phenotypic variation between siblings with Metachromatic Leukodystrophy. Orphanet J Rare Dis 2019;14(1):136. |
|  | 335 | Brown TM, Martin S, Fehnel SE, Deal LS. Development of the Impact of Juvenile Metachromatic Leukodystrophy on Physical Activities scale. Journal of Patient reported Outcomes 2017;2(1):15. |
|  | 668 | Kehrer C, Blumenstock G, Raabe C, Krageloh-Mann I. Development and reliability of a classification system for gross motor function in children with metachromatic leucodystrophy. Dev Med Child Neurol 2011;53(2):156-60. |
|  | 4090 | Bascou NA, Carson V, Safonova A, Poe MD, Escolar ML. A prospective natural history study of metachromatic leukodystrophy: A 20year study. Presented at the WORLDSymposium; United States. Mol Genet Metab 2020;129(2):129 (2) (pp S26-S27). |
|  | 4126 | Ammann L, Groschel S, Kehrer C, Frolich S, Krageloh-Mann I. Living with severe rare chronic neurological disease in childhood: Family burden and quality of life in families with MLD and PCH2. Presented at the 44th Annual Meeting of the Society for Neuropediatrics: Germany. Neuropediatrics 2018;49(Suppl 2). |
|  | 4127 | Amedick LB, Kehrer C, Beschle J, Strolin M, Wilke M, Wolf N, et al. Diffusion tensor imaging parameters in metachromatic leukodystrophy: Biomarkers for disease progress and therapeutically therapeutic evaluation. Presented at the 44th Annual Meeting of the Society for Neuropediatrics: Germany. Neuropediatrics 2018;49(Suppl 2). |
|  | 4170 | Bascou N, Poe M, Escolar M. Natural history of metachromatic leukodystrophy. Presented at the 13th International Congress of Inborn Errors of Metabolism (ICIEM 2017); Brazil. Journal of Inborn Errors of Metabolism and Screening 2017;5:327-328. |
|  | 4210 | Beschle J, Groeschel S, Kehrer C, Strolin M, Raabe C, Bayha U, et al. Haematopoietic stem cell transplantation in juvenile metachromatic leukodystrophy-What does the early course tell about long term outcome? Presented at the 12th European Paediatric Neurology Society Congress (EPNS 2017): France. Europ J Paediatr Neurol 2017;21(Suppl 1):e19. |
|  | 4291 | Carson VJ, Poe MD, Escolar ML. Natural history of metachromatic leukodystrophy. Presented at the 44th Annual Meeting of the Child Neurology Society; National Harbor: US. Ann Neurol 2015;78(SUPPL. 19):S192. |
|  | 4333 | Buhrman DZ, Lewis R, Poe M, Escolar ML. Natural history of progression of metachromatic leukodystrophy. Presented at the 10th Annual Research Meeting of the Lysosomal Disease Network (WORLD Symposium); San Diego: US. Mol Genet Metab 2014;111(2):S29. |
|  | 4440 | Kehrer C, Groschel S, Muller I, Krageloh-Mann I. Stem cell transplantation (SCT) in metachromatic leukodystrophy (MLD)-results from 9 patients. Presented at the 38th Annual Meeting of the Society of Neuropediatrics; Munster: Germany. Neuropediatrics 2012;43(2). |
|  | 4506 | Biffi A, Sessa M, Montini E, Naldini L. HSC gene therapy trial for metachromatic leukodystrophy. Presented at the European Society of Gene and Cell Therapy British Society for Gene Therapy Collaborative Congress; Brighton: UK. Hum Gene Ther 2011;22(10):A7-A8. |
|  | 4577 | Biffi A, Sessa M, Plati T, Lorioli L, Montini E, Benedicenti F, et al. HSC gene therapy trial for Metachromatic Leukodystrophy: First report on gene marking efficiency. Presented at the 18th Annual Congress of the European Society of Gene and Cell Therapy (ESGCT 2010); Milan: Italy. Hum Gene Ther 2010;21(10):1363. |
|  | 5860 | NCT02171104 MT2013-31: allo HCT for Metabolic Disorders and Severe Osteopetrosis. https://clinicaltrials.gov/show/NCT02171104 2014. |
|  | 6073 | Sessa M, Biffi A, Fumagalli F, Lorioli L, Plati T, Baldoli C, et al. Phase I/II Clinical Trial of Haematopoietic Stem Cell Gene Therapy For the Treatment of Metachromatic Leukodystrophy. In: European Neurological Society Annual Meeting 2011; 28-May-2011, 2011:S232-S234. Available from: European Neurological Society (ENS)  https://discovery.northernlight.com/document.php?datasource=PHE&docid=PE20121023040053650&context=WK%40northernlight.com  https://ovidsp.ovid.com/ovidweb.cgi?T=JS&CSC=Y&NEWS=N&PAGE=fulltext&D=dscv2&AN=PE20121023040053650 |
|  | 6106 | Yoon IC, Bascou NA, Poe MD, Escolar ML. Long-term neurodevelopmental, neurophysiological, and neuroradiological outcomes of hematopoietic stem cell transplantation for treatment of late-infantile metachromatic leukodystrophy. Mol Genet Metab 2020;129(2):S164-S165. |
|  | 6118 | Calbi V, Fumagalli F, Acquati S, Miglietta S, Ciotti F, Fraschini M, et al. Lentiviral haematopoietic stem cell gene therapy (HSC-GT) for metachromatic leukodystrophy (MLD): Preliminary results from a clinical trial with a cryopreserved formulation of OTL-200. Hum Gene Ther 2019;30(11):A146-A147. |
|  | 6156 | Calabria A, Spinozzi G, Rancoita P, Benedicenti F, Cesana D, Acquati S, et al. Characterization of Hematopoietic System Reconstitution In Vivo in Metachromatic Leukodystrophy Gene Therapy Patients. Molecular Therapy 2019;27(4):327-327. |
|  | 6187 | Calabria A, Spinozzi G, Rancoita P, Benedicenti F, Cesana D, Acquati S, et al. Characterization of hematopoietic system reconstitution in vivo in metachromatic leukodystrophy gene therapy patients. Hum Gene Ther 2018;29(12):A124-A125. |
|  | 6223 | Calabria A, Spinozzi G, Merelli I, Beretta S, Rancoita P, Benedicenti F, et al. Molecular Characterization of Hematopoietic System Reconstitution in Metachromatic Leukodystrophy Patients Following Hematopoietic Stem Cell Gene Therapy. Molecular Therapy 2018;26(5):312-313. |
|  | 6257 | Calbi V, Fumagalli F, Lorioli L, Sessa M, Bernardo ME, Cugnata F, et al. Update on safety and efficacy of lentiviral haematopoietic stem cell gene therapy (HSC-GT) for metachromatic leukodystrophy (MLD). Hum Gene Ther 2017;28(12):A67-A67. |
|  | 6728 | Biffi A, Sessa M, Capotondo A, Cesani M, Fasano S, Del Carro U, et al. Haematopoietic stem cell based gene therapy for the treatment of metachromatic leukodystrophy. Bone Marrow Transplant 2007;39:S36-S36. |
|  | 7268 | Orchard T, Ospedale San Raffaele - Telethon Institute for Gene T. OTL-200 in Patients With Late Juvenile Metachromatic Leukodystrophy (MLD). NCT04283227. In: ClinicalTrials.gov [Internet]. Bethesda (MD): National Library of Medicine (US). 2032 [accessed 19.5.20]. Available from: https://ClinicalTrials.gov/show/NCT04283227 |
|  | 7272 | Shenzhen Second People's H, Shenzhen U, Guangzhou W, Children's Medical C. Autologous Hematopoietic Stem Cell Gene Therapy for Metachromatic Leukodystrophy and Adrenoleukodystrophy. NCT02559830. In: ClinicalTrials.gov [Internet]. Bethesda (MD): National Library of Medicine (US). 2025 [accessed 19.5.20]. Available from: https://ClinicalTrials.gov/show/NCT02559830 |
|  | 7285 | University of P. The Natural History of Metachromatic Leukodystrophy. NCT00639132. In: ClinicalTrials.gov [Internet]. Bethesda (MD): National Library of Medicine (US). 2021 [accessed 19.5.20]. Available from: https://ClinicalTrials.gov/show/NCT00639132 |
| **The 2021 update** | 7338 | Morena F, Argentati C, Acquati S, DeWall S, Kelly F, Calbi V, et al. Toward reference intervals of ARSA activity in the cerebrospinal fluid: implication for the clinical practice of metachromatic leukodystrophy. J Appl Lab Med 2021;6(2):354-366. |
|  | 8114 | National Organization for Rare Disorders, Food and Drug Administration. The natural history of metachromatic leukodystrophy study (HOME Study). NCT04628364 In: ClinicalTrials.gov [Internet]. Bethesda (MD): National Library of Medicine (US). 2021 [accessed 12.7.21]. Available from: https://ClinicalTrials.gov/show/NCT04628364 |
|  | 8129 | Sohag University. Characterization and outcome of children with leukodystrophy: an observational study at Sohag University Hospital. NCT04781010 In: ClinicalTrials.gov [Internet]. Bethesda (MD): National Library of Medicine (US). 2021 [accessed: 12.7.21]. Available from: https://ClinicalTrials.gov/show/NCT04781010 |

### Table 15: List of studies excluded due to ‘Not relevant study design’ – 46 studies

|  | **Endnote #** | **Citation** |
| --- | --- | --- |
| **The 2020 original systematic review** | 53 | Mahmood A, Berry J, Wenger DA, Escolar M, Sobeih M, Raymond G, et al. Metachromatic leukodystrophy: a case of triplets with the late infantile variant and a systematic review of the literature. J Child Neurol 2010;25(5):572-80. |
|  | 68 | GlaxoSmithKline Group. Gene therapy protocol using autologous haematopoietic stem cells for MLD-C02, a patient with metachromatic leukodystrophy (MLD). 2018N356337_00/207394 [PDF provided by Orchard]: GlaxoSmithKline, 28th September 2019 [accessed 14.4.20]. 103p. |
|  | 99 | Beerepoot S, Nierkens S, Boelens JJ, Lindemans C, Bugiani M, Wolf NI. Peripheral neuropathy in metachromatic leukodystrophy: current status and future perspective. Orphanet J Rare Dis 2019;14(1):240. |
|  | 110 | Ashrafi MR, Amanat M, Garshasbi M, Kameli R, Nilipour Y, Heidari M, et al. An update on clinical, pathological, diagnostic, and therapeutic perspectives of childhood leukodystrophies. Expert Rev Neurother 2020;20(1):65-84. |
|  | 163 | Harrington M, Whalley D, Twiss J, Rushton R, Martin S, Huynh L, et al. Insights into the natural history of metachromatic leukodystrophy from interviews with caregivers. Orphanet J Rare Dis 2019;14(1):89. |
|  | 238 | Penati R, Fumagalli F, Calbi V, Bernardo ME, Aiuti A. Gene therapy for lysosomal storage disorders: recent advances for metachromatic leukodystrophy and mucopolysaccaridosis I. J Inherit Metab Dis 2017;40(4):543-554. |
|  | 260 | Saute JA, Souza CF, Poswar FO, Donis KC, Campos LG, Deyl AV, et al. Neurological outcomes after hematopoietic stem cell transplantation for cerebral X-linked adrenoleukodystrophy, late onset metachromatic leukodystrophy and Hurler syndrome. Arq Neuropsiquiatr 2016;74(12):953-966. |
|  | 310 | Chen X, Gill D, Shaw P, Ouvrier R, Troedson C. Outcome of Early Juvenile Onset Metachromatic Leukodystrophy After Unrelated Cord Blood Transplantation: A Case Series and Review of the Literature. J Child Neurol 2016;31(3):338-44. |
|  | 314 | Page KM, Stenger EO, Connelly JA, Shyr D, West T, Wood S, et al. Hematopoietic Stem Cell Transplantation to Treat Leukodystrophies: Clinical Practice Guidelines from the Hunter's Hope Leukodystrophy Care Network. Biol Blood Marrow Transplant 2019;25(12):e363-e374. |
|  | 318 | Harrington M, Hareendran A, Skalicky A, Wilson H, Clark M, Mikl J. Assessing the impact on caregivers caring for patients with rare pediatric lysosomal storage diseases: development of the Caregiver Impact Questionnaire. Journal of Patientreported Outcomes 2019;3(1):44. |
|  | 341 | Amin M, Elsayed L, Ahmed AE. Clinical and Genetic Characteristics of Leukodystrophies in Africa. J Neurosci Rural Pract 2017;8(Suppl 1):S89-S93. |
|  | 355 | Gulati S, Jain P, Chakrabarty B, Kumar A, Gupta N, Kabra M. The spectrum of leukodystrophies in children: Experience at a tertiary care centre from North India. Ann Indian Acad Neurol 2016;19(3):332-8. |
|  | 401 | Richards J, Korgenski EK, Srivastava R, Bonkowsky JL. Costs of the diagnostic odyssey in children with inherited leukodystrophies. Neurology 2015;85(13):1167-70. |
|  | 464 | Solders M, Martin DA, Andersson C, Remberger M, Andersson T, Ringden O, et al. Hematopoietic SCT: a useful treatment for late metachromatic leukodystrophy. Bone Marrow Transplant 2014;49(8):1046-51. |
|  | 482 | Musolino PL, Lund TC, Pan J, Escolar ML, Paker AM, Duncan CN, et al. Hematopoietic stem cell transplantation in the leukodystrophies: a systematic review of the literature. Neuropediatrics 2014;45(3):169-74. |
|  | 554 | Krageloh-Mann I, Groeschel S, Kehrer C, Opherk K, Nagele T, Handgretinger R, et al. Juvenile metachromatic leukodystrophy 10 years post-transplant compared with a non-transplanted cohort. Bone Marrow Transplant 2013;48(3):369-75. |
|  | 623 | Groeschel S, Kehrer C, Engel C, C ID, Bley A, Steinfeld R, et al. Metachromatic leukodystrophy: natural course of cerebral MRI changes in relation to clinical course. J Inherit Metab Dis 2011;34(5):1095-102. |
|  | 636 | Wang RY, Bodamer OA, Watson MS, Wilcox WR, Diseases AWGoDCoLS. Lysosomal storage diseases: diagnostic confirmation and management of presymptomatic individuals. Genet Med 2011;13(5):457-84. |
|  | 670 | Cable C, Finkel RS, Lehky TJ, Biassou NM, Wiggs EA, Bunin N, et al. Unrelated umbilical cord blood transplant for juvenile metachromatic leukodystrophy: a 5-year follow-up in three affected siblings. Mol Genet Metab 2011;102(2):207-9. |
|  | 687 | Smith NJ, Marcus RE, Sahakian BJ, Kapur N, Cox TM. Haematopoietic stem cell transplantation does not retard disease progression in the psycho-cognitive variant of late-onset metachromatic leukodystrophy. J Inherit Metab Dis 2010;33 Suppl 3:S471-5. |
|  | 696 | Bonkowsky JL, Nelson C, Kingston JL, Filloux FM, Mundorff MB, Srivastava R. The burden of inherited leukodystrophies in children. Neurology 2010;75(8):718-25. |
|  | 701 | Artigalas O, Lagranha VL, Saraiva-Pereira ML, Burin MG, Lourenco CM, van der Linden H, Jr., et al. Clinical and biochemical study of 29 Brazilian patients with metachromatic leukodystrophy. J Inherit Metab Dis 2010;33 Suppl 3:S257-62. |
|  | 788 | Pierson TM, Bonnemann CG, Finkel RS, Bunin N, Tennekoon GI. Umbilical cord blood transplantation for juvenile metachromatic leukodystrophy. Ann Neurol 2008;64(5):583-7. |
|  | 796 | Tokimasa S, Ohta H, Takizawa S, Kusuki S, Hashii Y, Sakai N, et al. Umbilical cord-blood transplantations from unrelated donors in patients with inherited metabolic diseases: Single-institute experience. Pediatr Transplant 2008;12(6):672-6. |
|  | 936 | Bindu PS, Mahadevan A, Taly AB, Christopher R, Gayathri N, Shankar SK. Peripheral neuropathy in metachromatic leucodystrophy. A study of 40 cases from south India. J Neurol Neurosurg Psychiatry 2005;76(12):1698-701. |
|  | 981 | Krivit W. Allogeneic stem cell transplantation for the treatment of lysosomal and peroxisomal metabolic diseases. Springer Semin Immunopathol 2004;26(1-2):119-32. |
|  | 991 | Ozkara HA, Topcu M. Sphingolipidoses in Turkey. Brain Dev 2004;26(6):363-6. |
|  | 1452 | Guffon N, Souillet G, Maire I, Dorche C, Mathieu M, Guibaud P. Juvenile metachromatic leukodystrophy: neurological outcome two years after bone marrow transplantation. J Inherit Metab Dis 1995;18(2):159-61. |
|  | 2477 | Haltia T, Palo J, Haltia M, Icen A. Juvenile metachromatic leukodystrophy. Clinical, biochemical, and neuropathologic studies in nine new cases. Arch Neurol 1980;37(1):42-6. |
|  | 4160 | Lum SH, Will A, Bonney D, Hiwarkar P, Poulton K, Church H, et al. A decade of low transplant-related morbidity and mortality in children with inherited metabolic diseases: A report from a single metabolic transplant centre in the Europe. Presented at the 2018 Blood and Marrow Transplantation Tandem Meetings (BMT 2018). Biol Blood Marrow Transplant 2018;24(3 Supplement 1):S423. |
|  | 4177 | Jalan A, Kudalkar K, Jalan R, Shinde D, Borugale M, Tawade R, et al. Spectrum of LSDS: An experience of 158 affected individuals from India. Presented at the 13th International Congress of Inborn Errors of Metabolism (ICIEM 2017); Brazil. Journal of Inborn Errors of Metabolism and Screening 2017;5:274-275. |
|  | 4383 | Groeschel S, Dali CI, Clas P, Kehrer C, Wilke M, Krageloh-Mann I. Cerebral gray and white matter volume changes and clinical course in metachromatic leukodystrophy (MLD). Presented at the 12th International Congress of the European Society of Magnetic Resonance in Neuropediatrics; Vienna: Austria. Neuropediatrics 2013;44(Suppl 1). |
|  | 4404 | Kehrer C, Groeschel S, Doering M, Krageloh-Mann I. 5-year follow-up in hematopoietic stem cell transplantation in 2 patients with late-infantile metachromatic leukodystrophy in comparison to an untreated cohort. Presented at the 10th European Paediatric Neurology Society Congress (EPNS 2013); Brussels: Belgium. Europ J Paediatr Neurol 2013;17(SUPPL. 1):S132. |
|  | 4444 | Feng X, Li C, Li N, Wu X. Clinical study of allogenetic hematopoietic stem cell transplantation for three cases of leukodystrophy. Presented at the 54th Annual Meeting of the American Society of Hematology (ASH 2012); Atlanta: US. Blood 2012;120(21). |
|  | 4601 | Smith A, Tolar J, Kivisto TJ, Lund T, Orchard P. Treatment of high risk inherited lysosomal and peroxisomal disorders using reduced intensity hematopoietic cell transplantation. Presented at the 51st Annual Meeting of the American Society of Hematology (ASH); New Orleans: US. Blood 2009;114(22). |
|  | 4700 | Kumar A, Narayanan K, Chaudhary RK, Mishra S, Kumar S, Vinoth KJ, et al. Current Perspective of Stem Cell Therapy in Neurodegenerative and Metabolic Diseases. Mol Neurobiol 2017;54(9):7276-7296. |
|  | 4825 | Wagemaker G. Lentiviral hematopoietic stem cell gene therapy in inherited immune and lysosomal enzyme deficiencies. Cell Ther Transplant 2016;5(4):56-62. |
|  | 4849 | Lorioli L, Biffi A. Hematopoietic stem cell transplantation for metachromatic leukodystrophy. Expert Opin Orphan Drugs 2015;3(8):911-919. |
|  | 5060 | Horwitz ME, Chao N. Umbilical cord blood transplantation for treatment of non-malignant disorders. Cell Ther Transplant 2010;2(7):no pagination. |
|  | 5111 | Prasad VK, Kurtzberg J. Umbilical cord blood transplantation for non-malignant diseases. Bone Marrow Transplant 2009;44(10):643-651. |
|  | 5227 | Rovelli AM, Steward CG. Hematopoietic cell transplantation activity in Europe for inherited metabolic diseases: Open issues and future directions. Bone Marrow Transplant 2005;35(SUPPL. 1):S23-S26. |
|  | 6239 | Harrington M, Whalley D, Twiss J, Rushton R, Martin S, Huynh L, et al. Metachromatic leukodystrophy and caregiver perspectives: Understanding the natural history of the disease from interviews with caregivers. Mol Genet Metab 2018;123(2):S60-S60. |
|  | 6274 | Adang LA, Sherbini O, Ball L, Bloom M, Darbari A, Amartino H, et al. Revised consensus statement on the preventive and symptomatic care of patients with leukodystrophies. Mol Genet Metab 2017;122(1-2):18-32. |
|  | 6553 | Sessa M, Biffi A, Fumagalli F, Lorioli L, Plati T, Baldoli C, et al. Phase I/II clinical trial of haematopoietic stem cell gene therapy for the treatment of metachromatic leukodystrophy. J Neurol 2011;258:232-233. |
|  | 6679 | Kehrer C, Kustermann-Kuhn B, Raabe C, Krageloh-Mann I. Natural history of metachromatic leucodystrophy (MLD) - clinical course. Eur J Pediatr 2008;167(3):374-374. |
|  | 7071 | Lockman LA, Shapiro EG, Krivit W. 10-year follow-up of bone-marrow transplantation for treatment of metachromatic leukodystrophy. Ann Neurol 1994;36(3):522-522. |

### Table 16: List of studies excluded due to ‘Duplicate’ – four papers (the 2020 original systematic review) and 22 studies (the 2021 update)

|  | **Endnote #** | **Citation** |
| --- | --- | --- |
| **The 2020 original systematic review** | 282 | Sessa M, Lorioli L, Fumagalli F, Acquati S, Redaelli D, Baldoli C, et al. Lentiviral haemopoietic stem-cell gene therapy in early-onset metachromatic leukodystrophy: an ad-hoc analysis of a non-randomised, open-label, phase 1/2 trial. Lancet 2016;388(10043):476-87. |
|  | 6454 | Boucher AA, Raymond GV, Shanley R, Orchard PJ, Miller WP. Outcomes Following Allogeneic Hematopoietic Stem Cell Transplantation For Metachromatic Leukodystrophy. Blood 2013;122(21):2. |
|  | 6504 | Singh J, Simmons L, Chakrapani A, Wassmer E. Metachromatic leukodystrophy: mortality data to support counselling of parents. J Inherit Metab Dis 2012;35:S107-S107. |
|  | 6581 | Bohringer J, Kustermann-Kuhn B, Gieseke F, Erbacher A, Doring M, Kehrer C, et al. Hematopoietic Stem Cell Therapy In Eight Patients with Metachromatic Leukodystrophy - Relevance of Post-Transplant Medication. Blood 2010;116(21):1529-1530. |
| **The 2021 update** | 7527 | Fumagalli F, Calbi V, Sessa M, Cugnata F, Rancoita PMV, Acquati S, et al. Lentiviral hematopoietic stem cell gene therapy (HSCGT) for metachromatic leukodystrophy (MLD) provides sustained clinical benefit. Bone Marrow Transplant 2019;54:76-7. |
|  | 7724 | Nafees B, de Freitas H, Lloyd A, Olaye A, Pang F. A societal utility study to elicit values for stages of metachromatic leukodystrophy (MLD) in the United Kingdom. Value Health 2020;23(Suppl 2):S713. |
|  | 7758 | Audouard E, Oger V, Meha B, Cartier N, Sevin C, Piguet F. Intravenous gene therapy approach for metachromatic leukodystrophy. Molecular Therapy 2021;29(4 Suppl 1):275. |
|  | 7828 |  |
|  | 7829 | Fumagalli F, Zambon AA, Rancoita PMV, Baldoli C, Canale S, Spiga I, et al. Metachromatic leukodystrophy: a single-center longitudinal study of 45 patients. J Inherit Metab Dis 2021;Epub ahead of print. |
|  | 7970 | Nafees B, de Freitas H, Lloyd A, Olaye A, Pang F. A societal utility study to elicit values for stages of metachromatic leukodystrophy (MLD) in the United Kingdom. Value Health 2020;23(Suppl 2):S713. |
|  | 7986 | Pang F, Dean R, Jensen I, Olaye A, Miller B. The cost-effectiveness of OTL-200 for the treatment of metachromatic leukodystrophy (MLD). Value Health 2021;24(Suppl 1):S203. |
|  | 8115 | Orchard Therapeutics, Ospedale San Raffaele. OTL-200 in patients with late juvenile metachromatic leukodystrophy (MLD). NCT04283227 In: ClinicalTrials.gov [Internet]. Bethesda (MD): National Library of Medicine (US). 2021 [accessed 12.7.21]. Available from: https://ClinicalTrials.gov/show/NCT04283227 |
|  | 8117 | Orchard Therapeutics, Ospedale San Raffaele. A safety and efficacy study of cryopreserved OTL-200 for treatment of metachromatic leukodystrophy (MLD). NCT03392987 In: ClinicalTrials.gov [Internet]. Bethesda (MD): National Library of Medicine (US). 2020 [accessed 12.7.21]. Available from: https://ClinicalTrials.gov/show/NCT03392987 |
|  | 8122 | Orchard Therapeutics, Ospedale San Raffaele. Gene therapy for metachromatic leukodystrophy (MLD). NCT01560182 In: ClinicalTrials.gov [Internet]. Bethesda (MD): National Library of Medicine (US). 2018 [accessed: 12.7.21]. Available from: https://ClinicalTrials.gov/show/NCT01560182 |
|  | 8126 | University of Pittsburgh. The natural history of metachromatic leukodystrophy. NCT00639132 In: ClinicalTrials.gov [Internet]. Bethesda (MD): National Library of Medicine (US). 2021 [accessed: 12.7.21]. Available from: https://ClinicalTrials.gov/show/NCT00639132 |
|  | 8145 | Orchard Therapeutics. OTL-200 in patients with late juvenile metachromatic leukodystrophy (MLD). NCT04283227 In: ClinicalTrials.gov [Internet]. Bethesda (MD): National Library of Medicine (US). 2020 [accessed: 12.7.21]. Available from: https://clinicaltrials.gov/show/NCT04283227 |
|  | 8148 | Orchard Therapeutics. A safety and efficacy study of cryopreserved OTL-200 for treatment of metachromatic leukodystrophy (MLD). NCT03392987 In: ClinicalTrials.gov [Internet]. Bethesda (MD): National Library of Medicine (US). 2017 [accessed: 12.7.21]. Available from: https://clinicaltrials.gov/show/NCT03392987 |
|  | 8153 | Orchard Therapeutics. Gene therapy for metachromatic leukodystrophy (MLD). NCT01560182 In: ClinicalTrials.gov [Internet]. Bethesda (MD): National Library of Medicine (US). 2012 [accessed: 12.7.21]. Available from: https://clinicaltrials.gov/show/NCT01560182 |
|  | 8157 | Fondazione Centro S. Raffaele Del Monte Tabor. A Phase I/II clinical trial of hematopoietic stem cell gene therapy for the treatment of metachromatic leukodystrophy - TIGET-MLD. EUCTR2009-017349-77-It. In: EU Clinical Trials Register (EUCTR) [Internet]. Amsterdam: European Medicines Agency (EMA). 2010 [accessed: 12.7.21]. Available from: https://www.clinicaltrialsregister.eu/ctr-search/search?query=eudract_number:2009-017349-77 |
|  | 8163 | National Organization for Rare Disorders. The natural history of metachromatic leukodystrophy study (HOME Study). NCT04628364 In: ClinicalTrials.gov [Internet]. Bethesda (MD): National Library of Medicine (US). 2020 [accessed: 12.7.21]. Available from: https://clinicaltrials.gov/show/NCT04628364 |
|  | 8165 | Shire. Natural history study of children with metachromatic leukodystrophy. NCT01963650 In: ClinicalTrials.gov [Internet]. Bethesda (MD): National Library of Medicine (US). 2013 [accessed: 12.7.21]. Available from: https://clinicaltrials.gov/show/NCT01963650 |
|  | 8169 | University of Pittsburgh. The natural history of metachromatic leukodystrophy. NCT00639132 In: ClinicalTrials.gov [Internet]. Bethesda (MD): National Library of Medicine (US). 2008 [accessed: 12.7.21]. Available from: https://clinicaltrials.gov/show/NCT00639132 |
|  | 8170 | Calbi V. Lentiviral haematopoietic stem cell gene therapy for metachromatic leukodystrophy: results in nine patients treated with a cryopreserved formulation of OTL-200 [ePoster 25]. Presented at WORLDSymposium 2021; 8-12 Feb 2021; Virtual Scientific Meeting. Mol Genet Metab 2021;132(2):2. |
|  | 8171 | Fumagalli F. Lentiviral hematopoietic stem and progenitor cell gene therapy  provides durable clinical benefit in early-symptomatic early-juvenile  metachromatic leukodystrophy. Presented at WORLDSymposium 2021; 8-12 Feb 2021; Virtual Scientific Meeting. Mol Genet Metab 2021;132(2):1. |
|  | 8172 | Fumagalli F. Lentiviral hematopoietic stem and progenitor cell gene therapy  provides durable clinical benefit in early-symptomatic early-juvenile  metachromatic leukodystrophy [ePoster 75]. Presented at WORLDSymposium 2021; 8-12 Feb 2021; Virtual Scientific Meeting. Mol Genet Metab 2021;132(2):1 |
|  | 8175 | Pang F. Health-related quality of life in metachromatic leukodystrophy based on a societal utility study in the UK [ePoster 187]. Presented at WORLDSymposium 2021; 8-12 Feb 2021; Virtual Scientific Meeting. Mol Genet Metab 2021;132(2):2. |

## References

[1] National Institute for Health and Care Excellence. *OTL-200 for treating metachromatic leukodystrophy: draft scope (pre-referral). Proposed Highly Specialised Technologies Evaluation [Word document provided by Orchard]*. London: NICE, November 2019 [accessed 14.4.20]. 6p.

[2] National Institute for Health and Care Excellence. *OTL-200 for treating metachromatic leukodystrophy [ID1666]: consultee and commentator comment form. Proposed Highly Specialised Technologies Evaluation [Word document provided by Orchard]*. London: NICE, n.d. [accessed 14.4.20]. 15p.

[3] Orchard Therapeutics. *2.7.3 summary of clinical efficacy [PDF provided by Orchard]*: Orchard Therapeutics, n.d. [accessed 14.4.20]. 228p.

[4] Orchard Therapeutics. *2.7.4 summary of clinical safety [PDF provided by Orchard]*: Orchard Therapeutics, n.d. [accessed 14.4.20]. 92p.

[5] Bley A, Muller I, Lobel U, Schrum J, Santer R, Hartmann M, et al. Hematopoietic stem cell transplantation (HSCT) in nine patients with juvenile MLD. Presented at the 39th Annual Meeting of the Society of Neuropediatrics; 25-28 April 2013; Innsbruck: Austria. *Neuropediatrics* 2013;44(2).

[6] Bohringer J, Kustermann-Kuhn B, Gieseke F, Erbacher A, Doring M, Kehrer C, et al. *Hematopoietic stem cell therapy in eight patients with metachromatic leukodystrophy relevance of post-transplant medication [Abstract 3727]. Presented at American Society of Hematology Annual Meeting and Exposition 2010; 4 Dec 2010; Orlando: US*, 2010

[7] Boucher AA, Miller W, Shanley R, Ziegler R, Lund T, Raymond G, et al. Long-term outcomes after allogeneic hematopoietic stem cell transplantation for metachromatic leukodystrophy: the largest single-institution cohort report. *Orphanet J Rare Dis* 2015;10:94.

[8] van den Broek BTA, Page K, Paviglianiti A, Hol J, Allewelt H, Volt F, et al. Early and late outcomes after cord blood transplantation for pediatric patients with inherited leukodystrophies. *Blood Adv* 2018;2(1):49-60.

[9] Groeschel S, Kuhl JS, Bley AE, Kehrer C, Weschke B, Doring M, et al. Long-term outcome of allogeneic hematopoietic stem cell transplantation in patients with juvenile metachromatic leukodystrophy compared with nontransplanted control patients. *JAMA Neurol* 2016;73(9):1133-40.

[10] Kehrer C, Groeschel S, Kustermann-Kuhn B, Burger F, Kohler W, Kohlschutter A, et al. Language and cognition in children with metachromatic leukodystrophy: onset and natural course in a nationwide cohort. *Orphanet J Rare Dis* 2014;9:18.

[11] Fumagalli F, Zambon AA, Rancoita PMV, Baldoli C, Canale S, Spiga I, et al. Metachromatic leukodystrophy: a single-center longitudinal study of 45 patients. *J Inherit Metab Dis* 2021;Epub 2021 Apr 15.

[12] Martin HR, Poe MD, Provenzale JM, Kurtzberg J, Mendizabal A, Escolar ML. Neurodevelopmental outcomes of umbilical cord blood transplantation in metachromatic leukodystrophy. *Biol Blood Marrow Transplant* 2013;19(4):616-24.

[13] Fumagalli F, Calbi V, Sora MGN, Sessa M, Baldoli C, Rancoita PMV, et al. Lentiviral haematopoietic stem-cell gene therapy for early-onset metachromatic leukodystrophy: long-term results from a non-randomised, open-label, phase 1/2 trial and expanded access. *Lancet* 2022;399(10322):372-83.

[14] Prasad VK, Mendizabal A, Parikh SH, Szabolcs P, Driscoll TA, Page K, et al. Unrelated donor umbilical cord blood transplantation for inherited metabolic disorders in 159 pediatric patients from a single center: influence of cellular composition of the graft on transplantation outcomes. *Blood* 2008;112(7):2979-89.

[15] Singh J, Simmons L, Chakrapani A, Wassmer E. Metachromatic leukodystrophy: mortality data to support counselling of parents. Presented at the Annual Symposium of the Society for the Study of Inborn Errors of Metabolism (SSIEM 2012); 4-7 Sept 2012; Birmingham: UK. *J Inherit Metab Dis* 2012;35(1 Suppl 1):S107.

[16] van Rappard DF, Boelens JJ, van Egmond ME, Kuball J, van Hasselt PM, Oostrom KJ, et al. Efficacy of hematopoietic cell transplantation in metachromatic leukodystrophy: the Dutch experience. *Blood* 2016;127(24):3098-101.

[17] Kehrer C, Blumenstock G, Gieselmann V, Krageloh-Mann I. The natural course of gross motor deterioration in metachromatic leukodystrophy. *Dev Med Child Neurol* 2011;53(9):850-5.
